# Developing Biomaterial‐Based mRNA Delivery System for Lung Disease Treatment

**DOI:** 10.1002/advs.202505413

**Published:** 2025-07-25

**Authors:** Qiancheng Gu, Huaqian Xue, Zhiyun Liu, Jiameng Rao, Lingyao Zeng, Chen Zhang, Lanjie Lei, Liyun Shi

**Affiliations:** ^1^ Key Laboratory of Artificial Organs and Computational Medicine in Zhejiang Province Institute of Translational Medicine Zhejiang Shuren University Hangzhou 310015 China; ^2^ Zhejiang Chinese Medical University Hangzhou 310053 China

**Keywords:** biomaterials, lung disease, mRNA, targeted therapy

## Abstract

Lung disease remains a persistent global health challenge. Advances in medical research have led to innovative strategies to combat these conditions, with biomaterials emerging as a promising platform for targeted drug delivery. Various biomaterials—including nanoparticles such as liposomes, polymers, hybrid systems, dendritic polymers, gold nanoparticles, mesoporous silica, calcium carbonate, and exosomes—exhibit excellent biocompatibility. These materials protect therapeutic agents from nuclease degradation, stabilize drug carriers, and enhance cellular uptake via mechanisms such as endocytosis. Chemical modifications further improve biomaterials by facilitating endosomal escape and conjugation with targeting ligands, thereby enabling precise delivery to specific cells or tissues. As a therapeutic modality, mRNA offers high biosafety, notable controllability, efficient translation, and immunomodulatory properties. This review evaluates the impact of lung structure on drug absorption, examines delivery mechanisms associated with various biomaterial types, and presents application examples. It also summarizes recent research developments, discusses clinical limitations, and explores future research directions for biomaterials in lung disease therapy. Additionally, it highlights the role of biomaterials in stabilizing and protecting mRNA, providing insights into the advancement of mRNA‐based therapeutics. This review aims to establish a robust theoretical foundation and offer practical guidance for biomaterial‐based mRNA therapies in treating lung diseases.

## Graphical abstract

Schematic illustration of biomaterial delivery mRNA for the treatment of lung diseases.

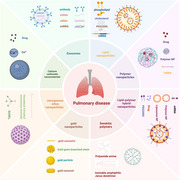

## 1 Introduction

Lung diseases, such as pneumonia, lung cancer, asthma, and pulmonary fibrosis, represent significant global health challenges. According to the International Respiratory Society Forum, pneumonia, lung cancer, chronic obstructive pulmonary disease (COPD), asthma, and tuberculosis are among the most prevalent lung diseases worldwide^[^ ^1^ ^]^ The World Health Organization states that chronic respiratory diseases are among the leading causes of mortality globally. affects millions, whereas asthma impacts ≈300–400 million people. Pneumonia remains the primary cause of death in children and the elderly, and tuberculosis results in ≈2 million fatalities each year. These conditions collectively contribute to a substantial disease burden and significant economic and social impacts.^[^ ^2^ ^]^ Current treatment strategies for lung diseases predominantly rely on pharmacological therapies, including bronchodilators,^[^ ^3^ ^]^ anti‐inflammatory drugs,^[^ ^4^ ^]^ and immunosuppressants.^[^ ^5^ ^]^ Although existing therapies have made some clinical progress, they still face significant clinical limitations, such as adverse drug reactions, potential risks of invasive procedures, and differences in individual efficacy. These challenges highlight the urgency of developing innovative treatments and improving the effectiveness of existing therapies.

mRNA‐based therapies present innovative strategies for lung disease treatment, offering a higher biosafety profile compared with conventional viral vectors due to their non‐integration into the host cell genome. In contrast to DNA‐based protein expression technologies, mRNA does not require entering the nucleus to function and, therefore, does not interact with the host genome.^[^ ^6^ ^]^ While mRNA translation has been successfully implemented in vivo, several challenges still exist, including the rapid degradation of mRNA prior to arriving at target cells, which is attributed to the action of ubiquitous extracellular ribonucleases. Additionally, the cell membrane hinders mRNA diffusion into the cytoplasm due to electrostatic repulsion, thereby limiting mRNA transfection. To address these challenges, researchers select appropriate targets, design and modify mRNA sequences to enhance stability,^[^ ^7^ ^]^ and employ tailored delivery platforms to ensure precise localization for accurate protein expression.^[^ ^8^ ^]^ Although mRNA stability and structural design are essential factors, the efficacy of the delivery system ultimately serves as the primary determinant of the therapeutic success of mRNA‐based therapies. Despite their numerous advantages, mRNAs are inherently fragile and require specific delivery strategies. By leveraging the engineering flexibility of biomaterials, mRNA payloads can be incorporated into nanoparticles that can prevent the triggering of unintended immune responses, target specific tissues, and efficiently deliver mRNA into the cytoplasm, thereby improving safety and bioactivity.

Without the protection and support of a delivery platform, mRNA alone has minimal therapeutic effect on the body. Various biomaterials have been developed to enhance mRNA delivery, including liposomes, polymer nanoparticles, hybrid nanoparticles, dendritic polymers, exosomes, and other nanoparticle systems. These materials facilitate the efficient delivery of mRNA, enhancing its functionality. Among these, the most commonly used are liposomal materials, which contain polar head groups, hydrophobic tails, and junctions.^[^ ^9^ ^]^ They engage with negatively charged mRNAs through electrostatic interactions, thereby safeguarding the functional integrity of mRNAs and promoting their escape from endosomes. Despite the limitations that challenges such as stability, delivery efficiency, and cell specificity have imposed on the clinical use of mRNA therapeutics, biomaterials as carriers offer significant protection against degradation. Lipid‐based materials such as lipid nanoparticles (LNPs) and lipid complexes can efficiently encapsulate mRNAs, protecting them from ribonuclease‐mediated degradation.^[^ ^10^, ^11^ ^]^ Moreover, specific polymers such as poly (beta‐amino esters) (PBAEs) can create stable complexes with mRNA, thereby improving its stability.^[^ ^12^ ^]^ The LNP‐based delivery systems can be significantly improved through formulation optimization. This includes fine‐tuning the weight ratio of the ionizable lipid C12‐200 to mRNA, adjusting the molar ratios of the individual components, and selecting the type and structure of phospholipids.^[^ ^13^ ^]^

Lung diseases frequently threaten health. mRNA therapies are emerging as promising solutions owing to their numerous advantages, but they rely heavily on effective delivery systems, with biomaterials serving as key carriers. At present, breakthroughs in this field remain scattered and lack systematic review. Previous literature often emphasizes either the characteristics of biomaterials or the principles of mRNA technology without exploring the integration of the two for lung diseases. Although earlier reviews addressed mRNA delivery or biomaterials, they rarely provided comprehensive and timely insights into combining the two for pulmonary disease treatment. Furthermore, most reviews do not incorporate new findings and recent data on this topic. The primary aim of this review is to explore the critical role of biomaterials in mRNA delivery and examine the properties of various biomaterials, including lipid nanoparticles, polymer nanoparticles, hybrid nanoparticles, exosomes, and other nanomaterials, along with their applications in lung disease treatment. Additionally, this paper highlights the advantages of exosomes in mRNA delivery, discusses recent research advancements, and presents practical applications in lung disease therapy. Through a detailed analysis of existing literature, this review comprehensively examines current advancements in biomaterial‐based delivery systems for treating lung diseases. Furthermore, it offers insights into future research directions, including developing innovative biomaterials, optimizing delivery systems, and potential pathways for clinical translation.

## 2 Basic Structure of the Lungs

**Figure 1:**
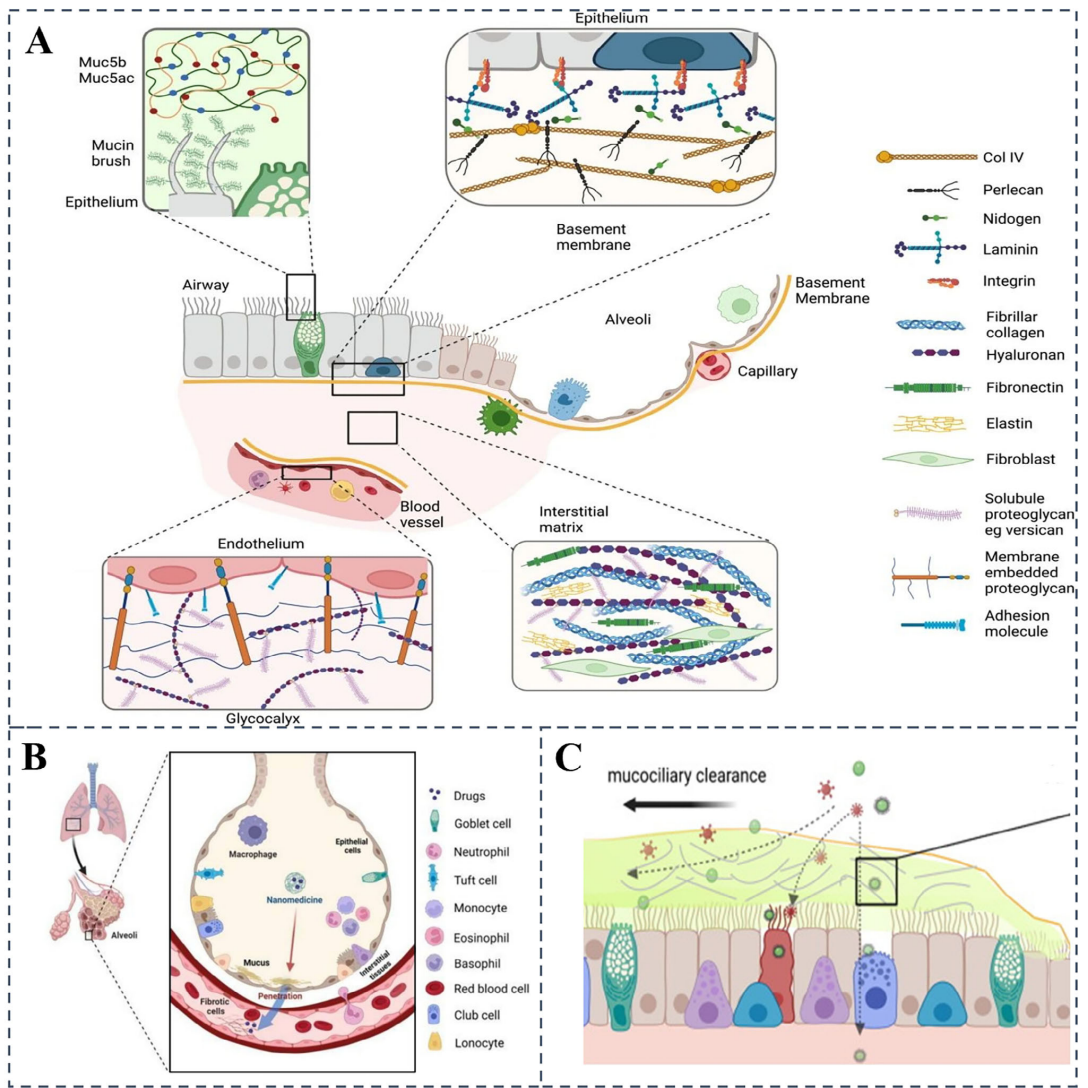
Lung structure diagram. A) Schematic representation of airway and alveolar structures in a healthy lung. Reproduced with permission.^[^ ^14^ ^]^ Copyright 2023 AAAS B) Inhalation‐mediated nanomaterial transport progresses sequentially through multiple biological barriers: traversing the mucus layer, penetrating alveolar epithelium and interstitial matrix, crossing capillary endothelium, dispersing into plasma, and finally interacting with erythrocytes in pulmonary capillaries. Reproduced with permission.^[^ ^15^ ^]^ Copyright 2023 Springer C) Airway mucus captures harmful particles (depicted as red spike structures) and therapeutic particles (depicted as green structures). Reproduced with permission.^[^ ^20^ ^]^ Copyright 2022 Elsevier.

The lungs, composed of a vast network of alveoli, are the most capillary‐rich organ in the human body^[^ ^14^ ^]^ (**Figure** **1**A). This structure enables rapid drug absorption following pulmonary administration, with low levels of chemical and enzymatic degradation, making the lung an ideal site for the delivery of bioactive macromolecules, such as proteins and nucleic acids. Drugs absorbed through the lungs can directly enter the bloodstream, bypassing the liver's first‐pass metabolism and enhancing bioavailability^[^ ^15^ ^]^(Figure 1B). The particle size of pulmonary inhalation formulations significantly influences their deposition location and form within the lungs.^[^ ^16^ ^]^ The alveolar surface fluid layer, type I alveolar cells, basement membranes, connective tissue layers, capillary basement membranes, and endothelium together form the “air–blood barrier.”^[^ ^17^ ^]^ Type I epithelial cells, which cover most of the alveolar surface area, offer an extensive area for drug contact and absorption, facilitating the relatively easy passage of drugs into the bloodstream. Additionally, receptors and transporter proteins on type I cells influence drug uptake and transport efficiency. Meanwhile, type II epithelial cells are essential for repairing and regenerating damaged alveolar epithelium.^[^ ^18^ ^]^

Pulmonary circulation is essential for delivering oxygen‐rich blood to tissues throughout the body, maintaining systemic oxygenation, and metabolic function. Systemic circulation, primarily confined to the large airways and pleura, supplies oxygenated blood to the pleura.^[^ ^19^ ^]^ Conversely, pulmonary circulation facilitates gas exchange by delivering deoxygenated blood to the lung parenchyma, with the lymphatic system complementing venous circulation. The respiratory tract, lined with a mucous membrane, serves as the passage for gases. The cilia on the respiratory mucosa expel mucus and foreign matter, aiding respiratory tract cleansing and protection (Figure 1C). The respiratory mucosa and the immune system resist pathogen invasion and prevent infections.^[^ ^20^, ^21^ ^]^ The trachea and bronchi form pathways for airflow, with their mucosal surfaces covered by cilia and mucus layers. This structure affects drug particle residence time and distribution in the airways.^[^ ^22^ ^]^ Drug deposition varies depending on dosage forms (e.g., aerosols, dry powder inhalers) and their interaction with the trachea and bronchi structures, impacting absorption efficiency. Conditions such as asthma, which involve airway narrowing due to smooth muscle spasms, further altering drug distribution and absorption.^[^ ^23^ ^]^

Alveolar fluid layers and surfactants influence drug stability and dispersion,^[^ ^24^ ^]^ whereas lung macrophages phagocytose foreign matter, including drug carriers, reducing the effective concentration and duration of drug action.^[^ ^25^ ^]^ Drug particles that are too large may be cleared by the mucus and cilia, whereas particles that are too small might be exhaled during expiration. For example, when administering liposomal drugs, the effects of alveolar surfactants on their stability must be considered; specific modifications may be required to improve their stability and targeting capabilities.^[^ ^26^ ^]^ Jiang et al. utilized ball milling to develop a new salvianolic acid (SAL) prescription incorporating l‐arginine and 2% lecithin.^[^ ^27^ ^]^ They reported that l‐arginine moderated the pronounced acidity of the SAL solution, whereas lecithin improved powder dispersibility and flowability.

## 3 Advantages of mRNA as a Treatment for Lung Diseases

### 3.1 Principles of mRNA Therapy

**Figure 2:**
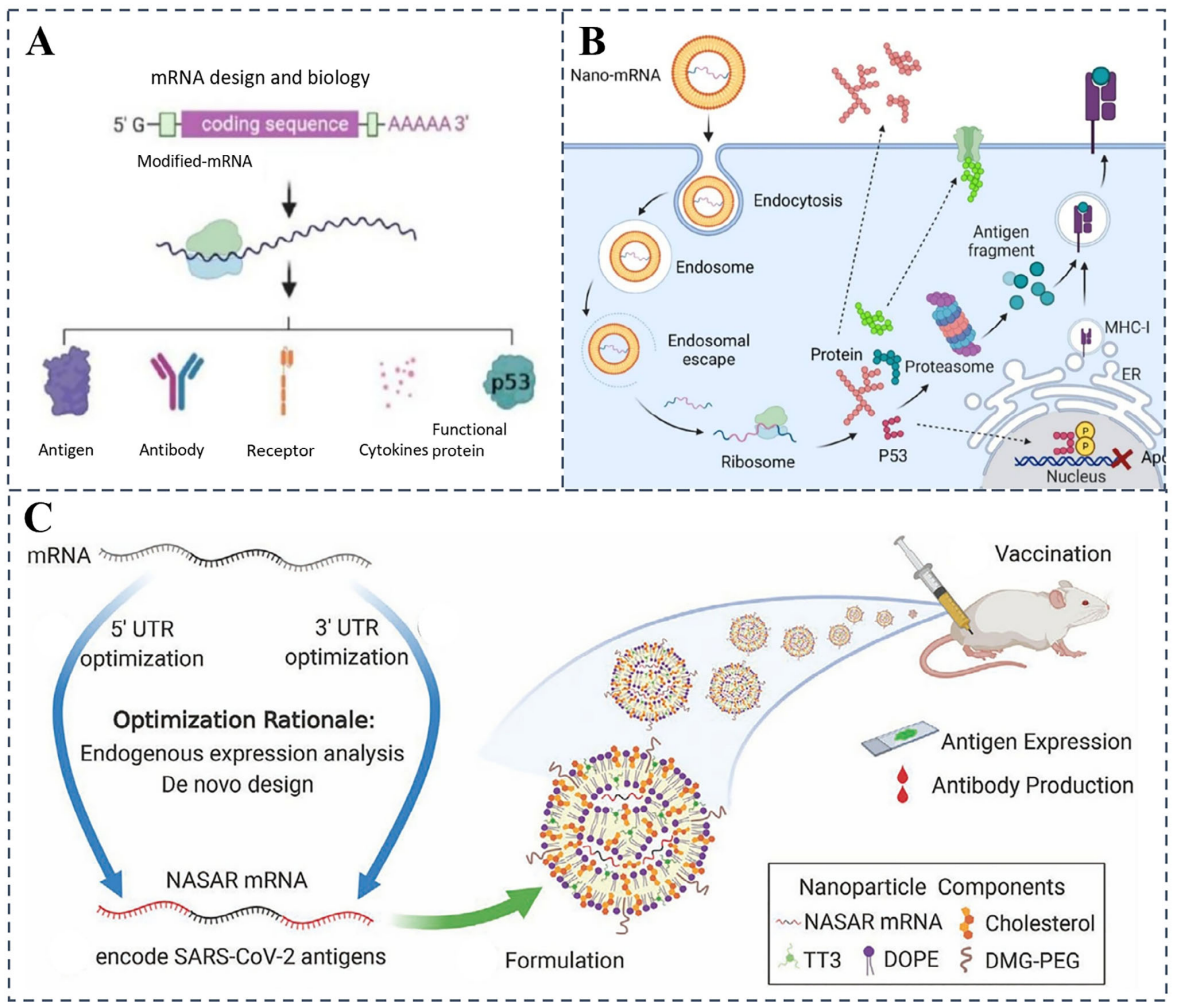
Principles of mRNA therapy. A) Basic principles of mRNA technology. B) Intracellular delivery and translation processes of mRNA encapsulated in nanomaterials. Reproduced with permission.^[^ ^28^ ^]^ Copyright 2023 Elsevier. C) Schematic illustration of the mRNA engineering process and its application as a potential SARS‐CoV‐2 vaccine. Reproduced with permission.^[^ ^29^ ^]^ Copyright 2020 Wiley.

mRNA, a nucleic acid molecule that carries genetic information, contains nucleotide sequences encoding specific proteins^[^ ^28^ ^]^ (**Figure** **2**A). Within the body, mRNA is rapidly engulfed by innate immune cells and subsequently degraded by nucleases. In contrast, mRNA‐encapsulated nanopreparations enter cells via the endocytic pathway and become trapped in acidic intracellular compartments. These nanopreparations typically facilitate the cytoplasmic release of mRNA by disrupting the endosomal or lysosomal membrane (Figure 2B). Through sequence design, mRNA can be engineered to produce therapeutically relevant proteins, such as those used in protein replacement therapies, transcriptional or growth factors for cellular reprogramming, or therapeutic antibodies for eliciting targeted immune responses in immunotherapies.^[^ ^10^ ^]^ Zeng et al. systematically modified the untranslated region (UTR) of mRNA to enhance protein expression and induce antigen‐specific antibody production at levels more than two orders of magnitude higher than those achieved using the FDA‐approved lipid nanoparticle material MC3 in vaccinated mice (Figure 2C).^[^ ^29^ ^]^ mRNAs can modulate the immune response through multiple mechanisms. For example, exogenously delivered mRNAs can mimic RNA viruses, leveraging their self‐adjuvant effects to stimulate pattern‐recognition receptors (PRRs) on cell surfaces or within endosomal compartments. These receptors include Toll‐like receptors (TLRs) 3, 7, and 8, as well as cytoplasmic RNA sensors such as retinoic acid‐inducible protein 1 (RIG‐1) and melanoma differentiation‐associated protein 5 (MDA5).^[^ ^30^ ^]^ Activation of these pathways enhances antigen‐presenting cell (APC) maturation, promotes inflammatory cytokine secretion, and improves adaptive lymphocyte function, ultimately shaping the immune response. The role of interferons (IFNs) in mRNA vaccines is intricate and highly context‐specific. IFN activation may lead to mRNA degradation and reduced antigen expression.^[^ ^31^ ^]^ In contrast, under specific conditions, well‐timed T‐cell receptor (TCR) activation with IFN signaling may enhance the immune response through TLR7‐mediated activation. Injection pathways also influence TCR activation and IFN signaling. For instance, subcutaneous or intramuscular injections may increase localized IFN expression in APCs, potentially inhibiting T‐cell activation. Conversely, intravenous injections can deliver mRNA‐lipid complexes directly to lymphoid organ APCs, enabling simultaneous IFN activation and TCR activation.^[^ ^32^ ^]^ Furthermore, the application of mRNA technology can be expanded to encode immunomodulatory molecules. This can be achieved by co‐encapsulating additional stimulatory molecules, such as CD40 ligand (CD40L), CD70, OX40 ligand (OX40L), or granulocyte–macrophage colony‐stimulating factor (GM‐CSF). RNA delivery vectors can be engineered to incorporate small molecules or lipid‐based adjuvants to modulate the immune response; these adjuvants can help adjust the immunogenicity of RNA formulations through pathways that do not involve IFN signaling.^[^ ^33^ ^]^

### 3.2 Advantages of mRNA Therapy

The potential of mRNA lies in its ability to drive the production of therapeutic proteins, enabling the treatment of a wide range of diseases across several critical areas. First, mRNA can restore the function of single proteins in rare monogenic diseases through protein replacement therapy. Second, it plays a vital role in cellular reprogramming by modulating cell behavior through the regulation of specific transcription factors or growth factors. Finally, in immunotherapy, mRNAs can encode transcripts that provoke an immune response specifically targeting designated cells, such as the production of therapeutic antibodies.^[^ ^34^ ^]^ The primary goal of mRNA‐based therapies is to facilitate the expression of therapeutic proteins meticulously engineered to exhibit minimal immunogenicity, prolonged stability, and enhanced translational efficacy. mRNA delivery technologies enable the expression of a diverse range of desired proteins in host cells and tissues while preserving the intrinsic capacity of host cells to conduct essential post‐translational modifications. This approach effectively addresses challenges associated with formulating and delivering protein‐based drugs, particularly those needed to restore intracellular function or transmembrane activities.^[^ ^35^ ^]^ Additionally, mRNA exhibits several notable advantages, including the reduced risk of insertional mutagenesis, the capacity for transient expression of encoded proteins, and the minimization of cellular barriers that may impede functional delivery. As mRNA operates primarily in the cytoplasm, these properties collectively establish it as a highly versatile and robust therapeutic tool.^[^ ^36^ ^]^

Although mRNA therapeutics hold immense potential, their efficacy is significantly limited without a suitable delivery vehicle. Optimization of delivery technologies is crucial for enhancing the in vivo stability and efficacy of mRNA‐based pharmaceuticals. The pharmacokinetics of mRNA drugs present six key challenges: (1) as a negatively charged macromolecule, mRNA has limited ability to cross the anionic cell membrane or enter cells through endocytosis^[^ ^37^ ^]^; (2) mRNA has a short intracellular half‐life and is highly sensitive to the almost ubiquitous RNA enzymes found in the cell; (3) even after internalization, the mRNA may fail to escape into the cytoplasm for translation; (4) the inherent structure of mRNA is immunogenic and can trigger toxic immune responses in vivo,^[^ ^38^ ^]^ (5) the large size of mRNA molecules limits their ability to cross the cell membrane, whereas instability limits intracellular applications; and (6) off‐target effects of mRNA molecules may lead to unintended consequences and potentially severe adverse effects.^[^ ^39^ ^]^ Biomaterial delivery systems offer effective solutions to these challenges, enabling mRNA drugs to achieve greater stability, targeted delivery, and improved therapeutic outcomes.

## 4 Biomaterials for mRNA Delivery

As a single‐stranded polynucleotide, mRNA is particularly susceptible to rapid degradation by extracellular ribonucleases before reaching target cells for transcription. Additionally, the electrostatic repulsion of the cellular membrane hinders mRNA diffusion into the cytoplasm, preventing successful transfection. Biomaterials provide essential physical and chemical protection for mRNA, safeguarding it from nuclease‐mediated degradation in vivo and ensuring its stability during delivery.^[^ ^40^ ^]^ Biomaterials with specific surface properties can be engineered to enhance interactions between mRNA carriers and cell membranes, thereby promoting cellular uptake through mechanisms such as endocytosis.^[^ ^41^ ^]^ Furthermore, biomaterials can be designed or modified to include components that disrupt endosomal membranes, thereby facilitating the escape of mRNA into the cytoplasm to undergo translation. Incorporating targeted ligands, such as antibodies, peptides, or small molecules, onto the surface of biomaterials enhances delivery specificity. This strategy allows selective delivery to specific cells or tissues, improves mRNA pharmacokinetics and biodistribution in vivo, and ensures an effective concentration of mRNA in target tissues.^[^ ^42^ ^]^ The release kinetics of mRNA can also be tailored through biomaterial design to achieve controlled release at specific times and locations, optimizing therapeutic efficacy.^[^ ^43^ ^]^ These advancements in biomaterials for mRNA delivery significantly enhance the stability, targeting, and overall effectiveness of mRNA‐based therapies.

### 4.1 Lipid Nanoparticles

**Figure 3:**
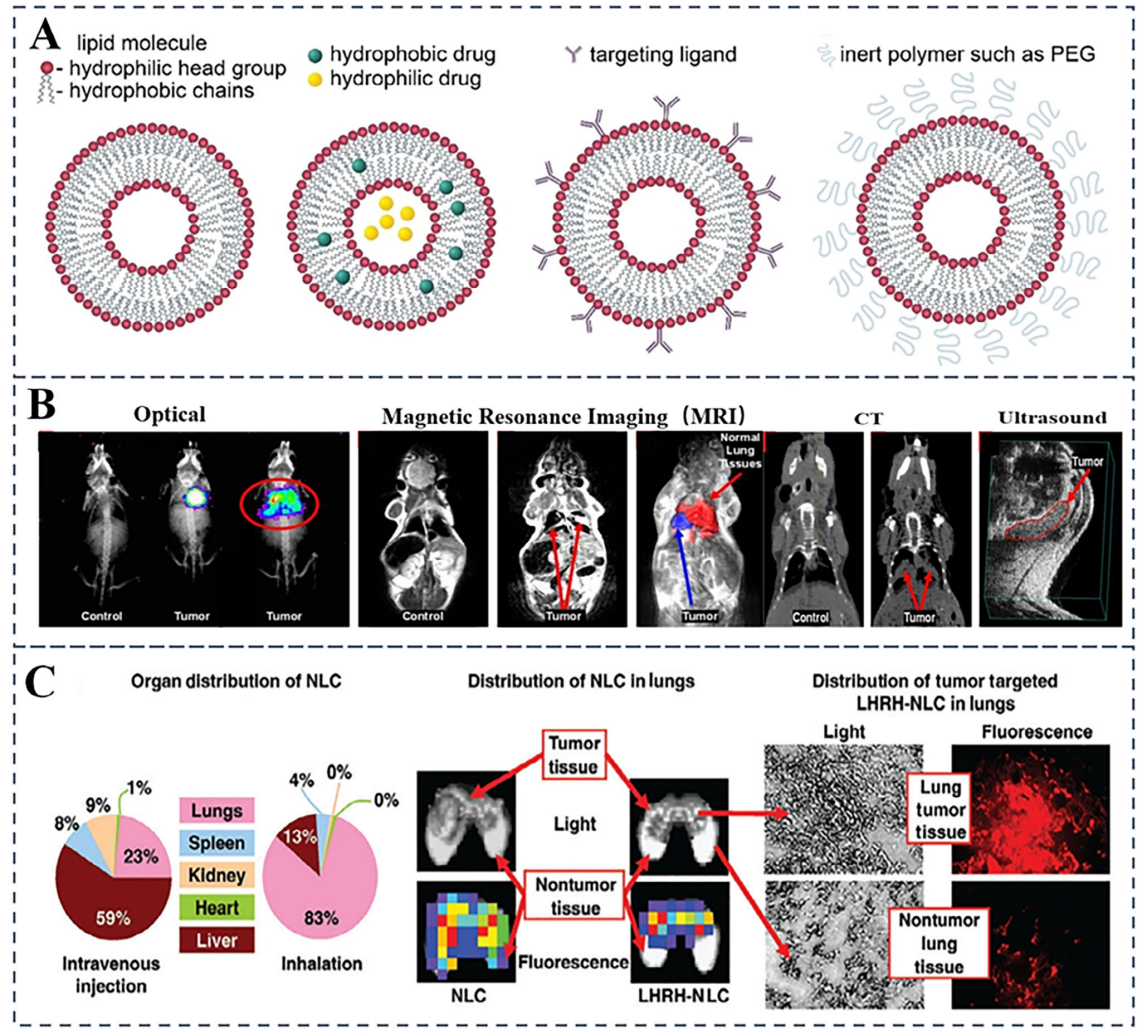
Illustration of a nanostructured lipid carrier‐based drug delivery system. A) Schematic representation of LNPs, LNPs encapsulating hydrophobic and hydrophilic drugs, immunoliposome functionalized with targeting ligands, and sterically stabilized (“stealth”) LNPS functionalized with inert polymers such as PEG. Reproduced with permission.^[^ ^64^ ^]^ Copyright 2021 Rumiana Tenchov. Published by American Chemical Society. B) Magnetic resonance imaging of control mice and mice with lung tumors of different sizes, and computed tomography images of control mice and mice with lung tumors. C) Localization of NLC within the lungs and its distribution in additional organs. Reproduced with permission.^[^ ^58^ ^]^ Copyright 2013 CRS.

Lipids are amphipathic molecules composed of three distinct structural domains: a polar head group, a hydrophobic tail region, and a linker that connects these two domains^[^ ^44^ ^]^ (**Figure** **3**A). LNPs are typically formulated using four essential components: ionizable cationic lipids, cholesterol, amphipathic phospholipids (PLs), and polyethylene glycol (PEG)‐conjugated lipids.^[^ ^45^ ^]^  PLs and cholesterol contribute to the structural stability of LNPs and influence endosomal escape by modulating membrane fluidity. PEG‐conjugated lipids inhibit aggregation and reduce macrophage‐mediated clearance, thereby enhancing the stability of the formulation.^[^ ^46^ ^]^ The surface properties and physicochemical characteristics of lipids can be altered through adjusting the ratio and structure of their components.^[^ ^47^ ^]^ For instance, ionizable cationic lipids are neutral at physiological pH, which reduces their interaction with serum proteins and clearance by macrophages. Upon delivered into an acidic intracellular endosomes or endolysosomes, the lipids become positively charged and escape from the lysosome to release mRNA release.^[^ ^48^ ^]^ Due to their nanometer size, LNPs can directly reach the lungs via the route of inhalation or intravenous, and subsequently distribute to lung tissues through the capillaries and their large surface areas. As drug carriers, LNPs provide several advantages, including high targeting efficiency, effective protection of encapsulated agents, controlled release for sustained therapeutic effects, substantial improvement in the therapeutic index, and reduced incidence of adverse drug reactions. Nanoparticles have been extensively applied in drug delivery and gene therapy.^[^ ^49^ ^]^ Common preparation methods include thin‐film hydration,^[^ ^50^ ^]^ supercritical fluid reverse‐phase evaporation,^[^ ^51^ ^]^ solvent injection,^[^ ^52^ ^]^ complex emulsion,^[^ ^53^ ^]^ and microfluidic technology.^[^ ^54^ ^]^

The physiological characteristics of the lungs make them an ideal site for LNPs accumulation. Due to their nanoscale particle size, LNPs can be effectively delivered directly to the lungs through inhalation, where they accumulate in lung tissues.^[^ ^55^ ^]^ This noninvasive delivery method provides direct access to epithelial cells in the airways, alveoli, and lung parenchyma, utilizing the lung's vast surface area and rich vasculature for rapid distribution.^[^ ^56^ ^]^ For instance, Sarode et al. developed a dry powder formulation of mRNA‐loaded LNPs for inhalation, optimizing excipients, lipid‐mRNA concentrations, and processing parameters.^[^ ^57^ ^]^ Intratracheal administration of this formulation demonstrated efficient lung deposition and transfection, ensuring successful delivery of functional mRNA. Surface modification of LNPs with antibodies or ligands targeting disease‐associated cells enhances specificity, increasing drug concentration at lesion sites and improving therapeutic efficacy. For example, Taratuia et al. designed a multifunctional nanostructured lipid carrier system (NLCS) for delivering anticancer drugs and siRNAs to the lungs through inhalation (Figure 3B,C).^[^ ^58^ ^]^

Ionizable lipids, which are neutral at physiological pH, minimize serum protein interactions and macrophage clearance but become positively charged in acidic environments (e.g., endosomes), promoting endosomal escape and releasing mRNA into the cytoplasm.^[^ ^54^ ^]^ A common example, DLin‐MC3‐DMA, is widely used in mRNA vaccine development.^[^ ^59^ ^]^ Cationic lipids tightly bind to negatively charged mRNAs through electrostatic interactions, improving encapsulation efficiency but often causing cytotoxicity and immunogenicity. For instance, DOTAP (1,2‐dioleoyl‐3‐trimethylammonium‐propane) is frequently employed in laboratory research as a cationic lipid for the delivery of mRNA.^[^ ^60^ ^]^ Cholesterol‐modified lipids increase stability and membrane rigidity, improving nanoparticle circulation and delivery efficiency.^[^ ^61^ ^]^ PEGylated lipids reduce nonspecific plasma protein binding, prolong circulation, and minimize macrophage‐mediated clearance. Optimization of LNP properties involves altering lipid type, proportion, and structure to improve interactions with cell membranes, cellular uptake, and endosomal escape.^[^ ^62^ ^]^ Additionally, PEG‐modified nanoparticles enhance circulation time by diminishing clearance mediated by renal excretion and the mononuclear phagocyte system (MPS) while also facilitating ligand conjugation for targeted delivery applications.^[^ ^38^ ^]^ Specific targeting ligands can be designed for markers such as sodium‐dependent phosphate transport protein 2b (NaPi2b), improving delivery precision to alveolar epithelial cells.^[^ ^63^ ^]^ As critical mRNA delivery carriers, LNPs provide effective protection, targeted transportation, and controlled release of mRNA by integrating and modifying their components. For lung delivery, their nanoscale size and specific surface properties enable efficient targeting and functionality at lesion sites.

Although LNPs are widely employed in mRNA vaccines (e.g., Pfizer/BioNTech), their large‐scale production requires microfluidic mixing devices to ensure consistent particle size and encapsulation efficiency. Scaling up this process while maintaining batch‐to‐batch uniformity remains a significant technical challenge. For instance, ionizable lipids (e.g., SM‐102) must be precisely mixed with mRNA at controlled ratios to prevent aggregation—a step that is difficult to automate for industrial‐scale manufacturing. Additionally, dependence on specialized lipids (e.g., DMG‐PEG) sourced from a limited number of suppliers increases production costs and introduces supply chain vulnerabilities. Future research should aim to develop novel LNPs formulations and preparation techniques to enhance delivery efficiency and safety.

### 4.2 Polymer Nanoparticles

Cationic polymers, a subset of polymers, are extensively employed as delivery vehicles for mRNA owing to their unique physicochemical properties, facilitating effective nucleic acid transport. These polymers can self‐assemble into nanoparticles by binding to negatively charged mRNA via electrostatic interactions. Polymer nanoparticles can be synthesized using either natural or synthetic materials, monomers, or preformed polymers, resulting in diverse structures and properties.^[^ ^65^ ^]^ The ability of polymers to protect mRNA, cellular uptake efficiency, and targeting can be optimized by adjusting their chemical structure, molecular weight, charge density, and surface functional groups. For instance, pH‐ or redox‐responsive polymeric materials have been developed to create the nanoparticles that can be triggered to release mRNA under specific lung microenvironmental conditions, such as acidic pH in tumor microenvironments or highly redox potentials at sites of inflammation.^[^ ^66^ ^]^ Additionally, modification of the surfaces of the nanoparticles with specific ligands or antibodies enables them to targeted delivery and binding to specific lung cell types. Various techniques, such as emulsification (through solvent displacement or diffusion),^[^ ^67^ ^]^ nanoprecipitation,^[^ ^68^, ^69^ ^]^ ionogelation,^[^ ^70^ ^]^ and microfluidization,^[^ ^71^ ^]^ are utilized in the synthesis of polymeric nanoparticles, each yielding distinct end products.

**Figure 4:**
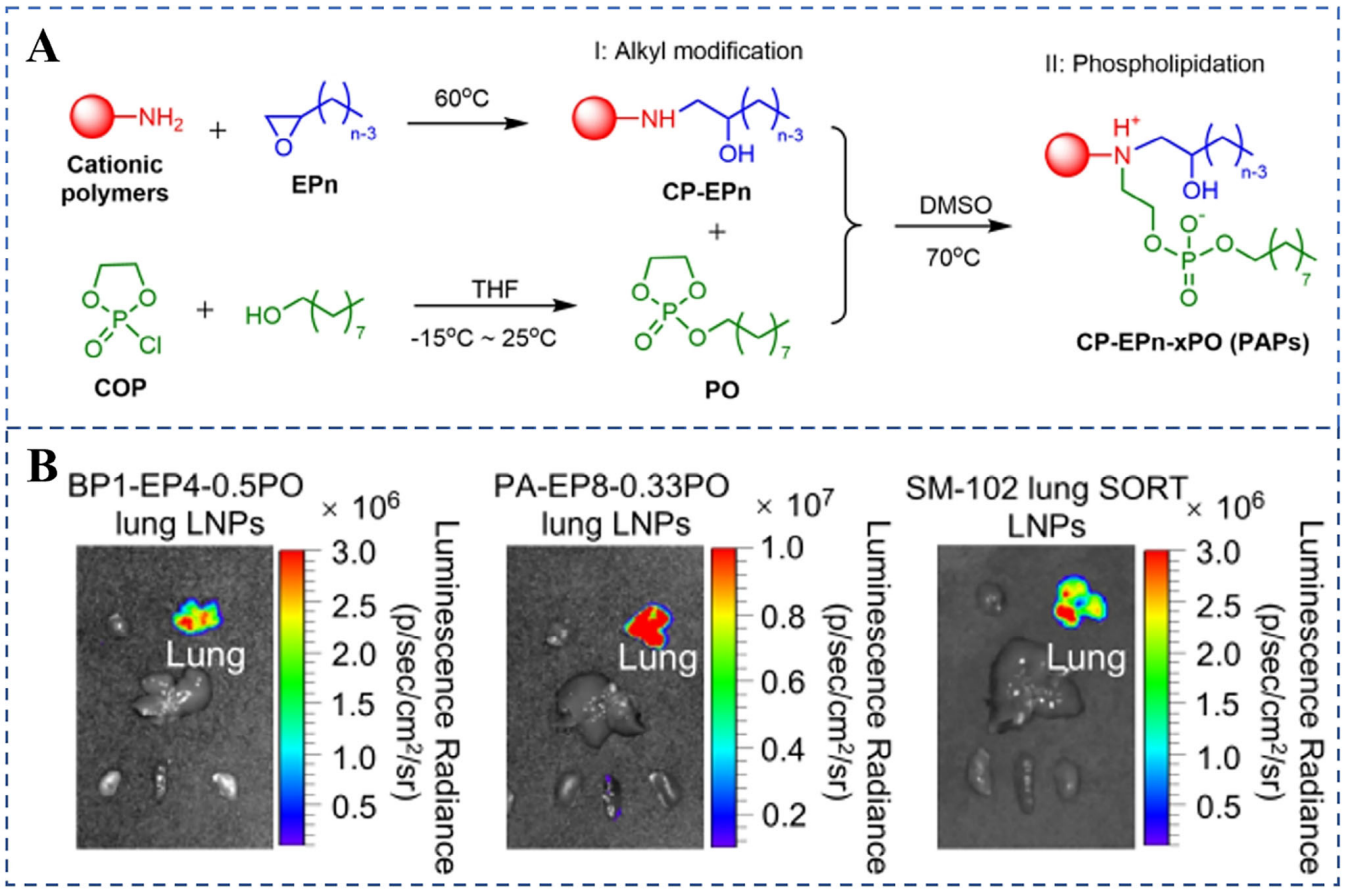
Conversion of cationic polymers into efficient and organ‐selective messenger ribonucleic acid (mRNA) carriers. A) The synthesis of PAPs. B) The translation of Fluc mRNA was visualized using three distinct lung‐targeted lipid nanoparticle formulations: BP1‐EP4‐0.5PO LNPs, PA‐EP8‐0.33PO LNPs, and SM‐102 SORT LNPs. Reproduced with permission.^[^ ^75^ ^]^ Copyright 2025 Angew Chem Int Ed Engl.

Polymeric nanoparticles are instrumental in facilitating the pulmonary delivery of mRNA. They provide a protective barrier against enzymatic degradation in the in vivo milieu, thereby safeguarding the stability and integrity of the mRNA molecules.^[^ ^72^ ^]^ Optimizing nanoparticle properties, such as size and surface charge, enhances mRNA uptake efficiency by lung cells.^[^ ^73^ ^]^ Additionally, carefully designed nanoparticle structures and compositions can enable slow and sustained mRNA release, prolonging its therapeutic action and minimizing unnecessary immune stimulation by lowering the immune response. Advances in polymeric materials have facilitated the creation of biocompatible and low‐toxicity options through novel material synthesis. Modifications with specific ligands or antibodies enable precise targeting of specific lung cell types.^[^ ^74^ ^]^ Understanding the distribution and metabolism of mRNA carried by polymer nanoparticles in the lungs is critical for optimizing delivery regimens. For instance, Lin et al. proposed a general strategy to convert cationic polymers into phosphorylated and alkylated polymers (PAPs) (**Figure** **4**A), which enables efficient and organ‐selective mRNA delivery in vivo and enhances delivery efficiency in the lungs (Figure 4B).^[^ ^75^ ^]^ Studies have shown that nanoparticles composed of polymers that carry mRNAs encoding tumor antigens activate antigen‐specific immune cells, thereby inducing long‐term antitumor immunity.^[^ ^76^ ^]^ Furthermore, pH‐responsive or redox‐responsive polymeric materials have been used to construct nanoparticles capable of releasing mRNA under specific lung microenvironmental conditions.^[^ ^77^, ^78^ ^]^ Implementing these strategies facilitates the accurate delivery of mRNA to the target site, thereby enhancing therapeutic efficacy while minimizing adverse effects on healthy tissues.

The diversity of polymer nanoparticles offers a wealth of options for mRNA delivery. Different synthesis methods yield nanoparticles with distinct characteristics, enabling customization for specific therapeutic needs. However, factors such as preparation complexity, cost, and effects on mRNA stability must be carefully considered when selecting a synthesis method. Similarly, choosing natural and synthetic materials requires balancing biocompatibility, degradability, and ease of functionalization. Polymeric nanoparticles have demonstrated substantial potential for lung‐specific mRNA delivery. Through targeted modification strategies and optimization of nanoparticle characteristics, researchers can significantly enhance the efficiency and specificity of mRNA delivery. The application of pH‐ and redox‐responsive materials provides innovative approaches for precision drug release. However, challenges remain, including the complexity of the lung microenvironment, individual variability in nanoparticle performance, and the need for further studies to evaluate long‐term antitumor immune effects and ensure safety and efficacy.

### 4.3 Lipid–Polymer Hybrid Nanoparticles

A novel generation of nanosystems combines the advantages of polymer and lipid‐based systems to form lipid–polymer hybrid nanoparticles (LPHNPs), which comprise a polymer core coated with a lipid shell.^[^ ^79^ ^]^ The polymer core is typically made of biodegradable and biocompatible materials, such as polylactic acid (PLA), polycaprolactone (PCL), polysaccharide F‐68, and chitosan.^[^ ^80^ ^]^ By integrating the biocompatibility and membrane fusion capabilities of lipids with the structural stability and modifiability of polymers, LPHNPs can effectively encapsulate a wide range of drugs, including small molecules, proteins, and nucleic acids, while achieving high drug‐loading capacities. They can be designed for sustained, slow, or stimuli‐responsive drug release, making them a promising solution for mRNA delivery. The composition of the polymer core can be tailored to optimize nanoparticle properties, such as biodegradability, stability, and drug release kinetics. These features, combined with the high drug‐loading capacity and stimulus‐responsive release, offer significant advantages for drug delivery systems.

**Figure 5:**
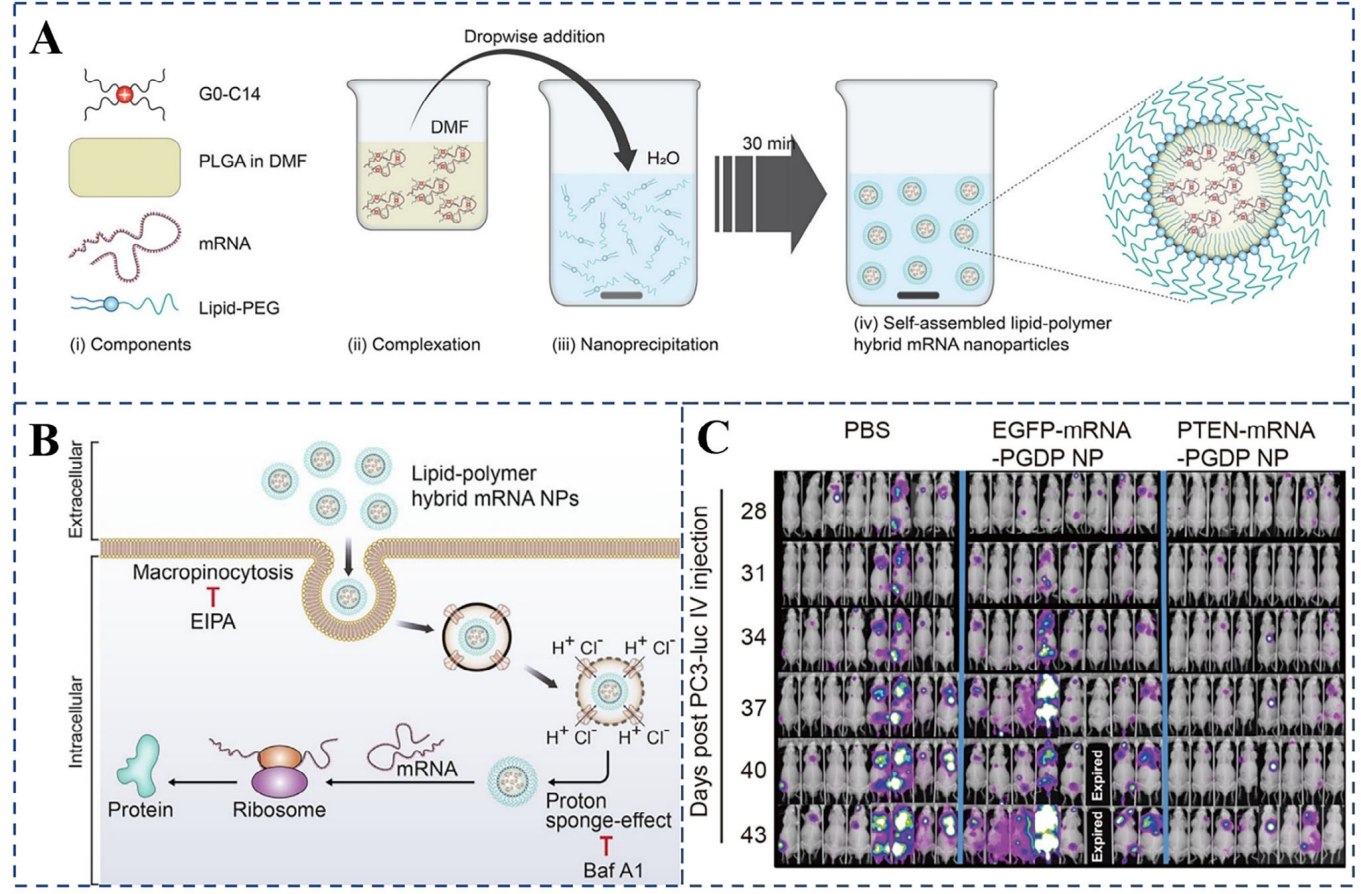
Preparation of mRNA nanoparticles (NPs). A) Diagram of the self‐assembly process and nanoparticle structure of polymer‐lipid hybrid mRNA NPs (i–iv). B) The cellular uptake and intracellular transport mechanisms of the hybrid mRNA nanoparticles are depicted schematically. C) Bioluminescent imaging was employed to evaluate the metastatic spread of PC3‐luc tumors at various time points following treatment. Reproduced with permission.^[^ ^81^ ^]^ Copyright 2018 Springer.

LPHNPs offer enhanced stability for mRNA by shielding it from degradation during delivery. For example, Islam et al. designed LPHNPs (**Figure** **5**A,(i–iv)) that extended the circulation time of mRNA following intravenous administration and prevented its uptake by the reticuloendothelial system, thus preserving the integrity of the mRNA and achieving tumor treatment effect (Figure 5B).^[^ ^81^ ^]^ LPHNPs can also cross the alveolar barrier due to their unique structure, enabling efficient delivery of mRNA into alveolar cells^[^ ^42^ ^]^ (Figure 5C). Optimizing nanoparticle size and surface properties can further enhance endocytosis by alveolar cells, increasing the intracellular delivery of mRNA. Guo et al. have successfully developed hybrid nanoparticles that feature a PLGA polymer core and a lipid shell modified with transferrin (Tf), thereby demonstrating the feasibility of this approach.^[^ ^82^ ^]^ These nanoparticles, which utilized TfR‐mediated endocytosis, were 2.8–4.1 folds more effective at internalization by A549 cells compared with PLGA‐only nanoparticles. LPHNPs provide robust protection for mRNA against enzymatic degradation in the lungs, maintaining its structural integrity and biological activity. Additionally, LPHNPs can withstand the complex microenvironment of the lungs, such as varying pH levels and redox states, ensuring stable mRNA encapsulation and release. By reducing immune responses and enhancing nanoparticle retention, they improve the stability and therapeutic effectiveness of mRNA in lung tissues. PEGylation increases nanoparticle hydrophilicity and stability, reduces nonspecific interactions, and prolongs circulation time in vivo.^[^ ^83^ ^]^ Cationic lipids or polymers enhance the efficiency of mRNA encapsulation by binding to negatively charged mRNAs through electrostatic interactions. Additionally, surface modifications with ligands, including antibodies, peptides, or folic acid, facilitate targeting specific cell receptors, improving delivery precision.^[^ ^84^ ^]^ For example, nanoparticles modified with tumor‐associated antigen antibodies can aggregate specifically at tumor sites, enhancing therapeutic efficacy.^[^ ^85^ ^]^ Stimulus‐responsive designs enable precise drug release under specific conditions, such as pH, redox environment, or enzymatic activity. Zhang et al. developed a pH‐responsive drug delivery system that targeted inflamed lung tissues in mice, triggering drug release in low‐pH environments following intravenous injection.^[^ ^86^ ^]^

In conclusion, LPHNPs exhibit remarkable performance in mRNA delivery, offering improvements in stability, targeting, and precision through various modification strategies. However, practical applications must consider the feasibility and safety of these modifications. For instance, stimulus‐responsive systems require further investigation into the regularity of lung‐specific stimuli to ensure timely and localized drug release. Similarly, advancements in precision targeting must focus on improving specificity and effectiveness to achieve optimal therapeutic outcomes.

### 4.4 Dendritic Polymers

Dendritic macromolecules are a unique class of polymers distinguished by their highly branched dendritic architecture. This structure has three main components: a central core, a polymer backbone, and numerous side chains. These include dendritic polyethyleneimine (PEI), poly (beta‐amino ester) (PBAE), poly‐l‐lysine (PLL), polypropyleneimine (PPI), polyamide‐amine (PAMAM), and ionizable amphiphilic Janus dendrimers (IAJD), among others. PAMAM dendrimers have been the most extensively investigated for their potential in mRNA delivery applications. Their core contains hydrogen bonds, amide groups, and tertiary amine groups, which enable them to bind to mRNA and form stable nanoparticles. However, the highly positive charge of tertiary amine end groups can lead to cytotoxicity, necessitating chemical modifications to reduce toxicity, enhance circulation time, or improve targeting capabilities. For instance, Chahal et al. designed a single‐dose, adjuvant‐free nanovaccine platform that employs alkyl chain‐modified PAMAM dendrimers to effectively encapsulate antigenic mRNAs, potentially treating various diseases.^[^ ^87^ ^]^

Dendritic polymers, with their highly branched structures, well‐defined compositions, and diverse functional groups, are gaining attention for biomedical applications.^[^ ^88^ ^]^ Their precise nanostructure offers a high specific surface area and abundant internal cavities, enhancing their adsorption and encapsulation capabilities. However, their potential for mRNA delivery remains largely unexplored. Notably, Chahal et al. created a fast‐response, fully synthesized dendritic polymer nanoparticle vaccine platform encoding antigens with encapsulated mRNA replicons.^[^ ^87^ ^]^ This system demonstrated protective immunity against many lethal pathogens, including H1N1 influenza, *Toxoplasma gondii*, and Ebola.^[^ ^89^ ^]^ Joubert et al. effectively achieved low toxicity and efficient mRNA delivery by chemically modifying PAMAM dendrimers with lysine as a site‐specific anchor. Incorporating functional groups into PAMAM and lysine‐based dendrimers has improved RNA condensation, fusogenicity, and buffering capacity.^[^ ^90^ ^]^ Combined with introducing fusion‐inducing moieties, these modifications improve endosomal escape, enabling cellular mRNA delivery and translation. Another promising approach involves dendrimer–lipid hybrid systems. Cheng et al. developed such a system using a self‐developed dendrimer (5A2‐SC8) combined with cholesterol, auxiliary phospholipids, and DMG‐PEG. This hybrid system demonstrated efficient and safe mRNA delivery.^[^ ^13^ ^]^ Meshanni et al. established a technique to induce transient protein expression of the anti‐inflammatory cytokine mRNA, TGF‐β, in the lower lung region.^[^ ^91^ ^]^ A single‐component ionizable amphiphilic Janus dendritic polymer was employed as the delivery vehicle, enabling accurate, efficient, and safe delivery to the mouse lung. This delivery system demonstrates strong potential for tissue‐specific targeting and presents a promising strategy to address the current clinical gap in treating parenchymal lung injury and disease. Zhu et al. developed a single‐component, multifunctional IAJD delivery platform.^[^ ^92^ ^]^ Synthesizing six IAJD libraries and modifying their sequences, they assembled dendrimer‐like nanoparticles (DNPs) with organ‐specific targeting capabilities to deliver precise mRNA. This strategy has shown greater efficiency and improved delivery performance compared to conventional lipid nanoparticle (LNP) systems, offering a more advanced approach for mRNA therapeutics.

Although dendritic polymers face challenges as standalone mRNA delivery systems, including cytotoxicity, recent research shows promise. Innovations such as chemical modification to reduce toxicity and hybridization with lipid systems enhance their potential to overcome these limitations. Future research must focus on analyzing the effect of specific chemical modifications on dendrimers' physicochemical and delivery‐related properties. Additionally, the long‐term stability of dendrimer–lipid hybrid systems and their adaptability to complex in vivo environments require further investigation. Improved integration of dendrimers with other delivery materials or technologies could unlock their full potential, ensuring their pivotal role in advancing mRNA delivery systems.

### 4.5 Gold Nanoparticles

Gold nanoparticles (AuNPs) are precious metal nanomaterials with exceptional biocompatibility, ease of surface modification, and unique optical properties, making them a promising material for disease treatment. They exhibit robust near‐infrared (NIR) absorption and high photothermal conversion efficiency, which render them ideal candidates for use as photoacoustic and photothermal agents in both photoacoustic imaging (PAI) and photothermal therapy.^[^ ^93^ ^]^ When combined with lipid nanoparticles, AuNPs act as contrast agents for in vivo imaging, enabling real‐time monitoring of drug distribution and release. Zu et al. successfully synthesized gold nanostars capped with BSA, with an average diameter of 85 nm, which exhibited superior biocompatibility and significantly enhanced photoacoustic (PA) signal intensity in HepG2 cells.^[^ ^94^ ^]^ Moreover, the nanostars improved the accuracy of computed tomography (CT) imaging, serving as a dual‐modal CT/PA imaging contrast agent. The mRNA delivery proved significantly more effective than that of DNA, thus providing the foundation for the delivery of mRNA by gold nanoparticles. Gu et al. devised a non‐cationic, multifunctional nanoparticle (NP) platform termed mRNA‐MPN NPs.^[^ ^95^ ^]^ The design of this platform rests on three main principles. First, the polydentate nature of polyphenols enables efficient mRNA doping. Second, metal–phenolic liganding offers abundant building blocks and diverse chemistry for NP construction. Third, a seeding agent can elevate the localized concentration of precursors (mRNA and polyphenol), promoting NP formation under ambient conditions. This platform is capable of efficient mRNA transfection. Its organophilicity is tunable, and it can deliver mRNA to multiple organs following intravenous injection. Given its non‐cationic nature, excellent biocompatibility, high mRNA delivery efficiency, and versatile modularity, mRNA‐MPN NPs present promising alternatives to current mRNA delivery systems. They also pave the way for the development of future NP therapeutic agents and hold potential for AuNPs‐mediated mRNA delivery in lung disease treatment. Despite the absence of gold nanoparticles that are engineered to deliver mRNA for the treatment of lung diseases, the aforementioned research establishes a foundational framework for future studies in this domain.^[^ ^96^ ^]^

Gold nanoparticles show significant potential as carriers for drug and gene delivery and in photothermal and photodynamic therapies. Integrating these materials with lipid nanoparticles presents novel strategies for enhancing drug delivery and facilitating in vivo imaging. AuNPs face two significant challenges: high production costs and concerns regarding heavy metal toxicity. The synthesis of monodisperse AuNPs with precise size and surface characteristics—such as gold nanorods or nanostars—often requires costly reagents (e.g., cetrimonium bromide) and tightly controlled laboratory conditions, making large‐scale production difficult. Furthermore, the potential for long‐term accumulation of gold in organs such as the liver and spleen raises significant safety concerns, particularly with repeated dosing. Although surface modifications, such as PEGylation, can help mitigate toxicity, they also introduce additional complexity and cost into the production process.

### 4.6 Mesoporous Silica Nanoparticles

According to the definition provided by the International Union of Pure and Applied Chemistry (IUPAC), mesoporous materials are characterized by pore diameters that fall within the range of 2–50 nm. Mesoporous silica nanoparticles (MSNs), a class of mesoporous material, have garnered significant research attention recently due to their uniform and tunable pore size, adjustable particle diameter, high surface area, and excellent biocompatibility.^[^ ^97^ ^]^ With the conjugation of targeting groups on their surfaces (e.g., antibodies, peptides, folic acid, etc.), MSNs are capable of targeted recognition and binding to specific cells or tissues. Additionally, MSNs' pore structure can be used to load mRNA and achieve controlled mRNA release with appropriate pore modification or capping strategies. These properties render MSNs highly stable and suitable for drug delivery carriers, particularly for precisely delivering therapeutic agents to lung lesions in treating lung diseases.

MSNs, as inorganic nanoparticles, have been comprehensively designed to optimize therapeutic delivery. Dong et al. developed a mesoporous silica nanoparticle (MSNP) system specifically designed for mRNA delivery.^[^ ^98^ ^]^ In this approach, mRNA was complexed with cationic polymers and subsequently bound to the MSNP surface via electrostatic interactions, employing a unique assembly method. The study also investigated the influence of nanoparticle size, porosity, surface properties, and shape on delivery efficiency. The optimized vector demonstrated superior performance for delivering luciferase mRNA in mice, exhibiting strong cellular uptake, efficient endosomal escape, and tissue‐specific expression. Notably, this vector achieved comparable efficacy in both murine and rat models, with no significant toxicity observed. The efficacy of the MSNP system has prompted further research in the field of molecular biology. Similarly, based on the concept of a silicon dioxide nanostructure, Zhang et al. constructed a subcutaneous delivery system based on mesoporous silica nanoparticles loaded with mRNA (MSN–mRNA).^[^ ^99^ ^]^ The formulation included unmodified mRNA and a subcutaneous depot of the imidazole‐oxindole PKR inhibitor C16. During transfection, C16 treatment emerged as an effective strategy for immune evasion. It nonlinearly enhanced the translation of unmodified mRNA in mouse fibroblasts and dendritic cells in vitro, outperforming nucleoside‐modified mRNA. C16 also further promoted the translation of nucleoside‐modified, HPLC‐purified mRNA. These findings highlight the strong translational potential of the MSN–mRNA delivery platform for mRNA‐based therapies.

Xian et al. synthesized a novel silica nanoparticle (SNP–CaClOH) functionalized with CaClOH and featuring a spiked surface morphology for mRNA delivery.^[^ ^100^ ^]^ This nanoparticle was produced through a carefully designed thermal decomposition process of water‐containing CaCl₂ within the silanol‐rich mesopores of preformed spiked SNPs. The inclusion of CaClOH imparted alkaline properties to the carrier, facilitating endosomal escape via the proton‐sponge effect. Moreover, SNP–CaClOH increased intracellular Ca^2^⁺ levels, which interact with calmodulin (CaM), to activate the mammalian target of rapamycin complex 1 (mTORC1), thereby enhancing mRNA translation. The spiked nanostructure further contributed to delivery efficiency, demonstrating robust performance in both in vitro and in vivo models. This positions SNP–CaClOH as a promising candidate for advancing mRNA delivery technologies, particularly in treating lung diseases.″ Despite their high drug‐loading capacity, MSNs present several manufacturing challenges. Inorganic synthesis methods, such as the Stöber process, generate significant chemical waste and require stringent control of pH and temperature to form uniform pore structures. Scaling these processes often leads to heterogeneous particle sizes and collapsed pores, diminishing mRNA encapsulation efficiency. Additionally, the nondegradable nature of silica raises long‐term biocompatibility concerns, necessitating surface modifications, such as organic polymer coatings, that further complicate large‐scale production.

However, practical applications of MSNs require further optimization of preparation methods to improve their stability and drug‐loading capacity. Moreover, additional research is necessary to evaluate their safety and efficacy for human use. With these advancements, MSNs could significantly improve therapeutic outcomes in pulmonary diseases.

### 4.7 Calcium Carbonate Nanomaterials

Calcium carbonate (CaCO₃) has gained significant attention in biomedical applications owing to its availability, low cost, safety, biocompatibility, pH sensitivity, and slow biodegradability.^[^ ^101^ ^]^ CaCO₃ nanoparticles hold significant potential as drug carriers, particularly for targeting cancerous tissues and cells. Their pH‐dependent properties and capacity for functionalization with targeting agents make them ideal candidates for anticancer drug delivery systems. Additionally, the slow degradation of CaCO₃ in the matrix allows these nanoparticles to act as sustained release systems, retaining drugs within cancerous tissues for extended periods post‐administration. These properties make calcium carbonate nanocarriers highly effective for controlled and sustained drug release.^[^ ^102^ ^]^

**Figure 6:**
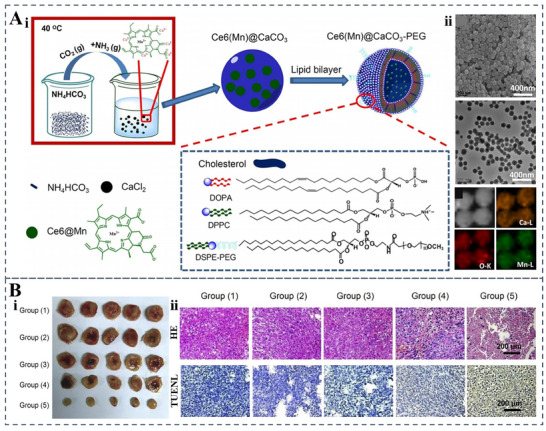
Synthesis and characterization of Ce6(Mn)@CaCO₃‐PEG nanoparticles. A) Synthesis and Structure diagram of Ce6(Mn)@CaCO3‐PEG nanoparticles (i). SEM and TEM images of Ce6(Mn)@CaCO_3_ nanoparticles, showing Ce6(Mn)@CaCO3 with calcium K edge (yellow), oxygen L edge (red), and manganese L edge (green) (ii). B) Tumor photographs(i) and H&E‐stained tumor sections were collected from all different groups of mice at the end of treatment(ii). Reproduced with permission.^[^ ^104^ ^]^ Copyright 2016 Elsevier.

Calcium carbonate nanomaterials offer good biocompatibility, possess a large specific surface area, and feature high porosity. These characteristics enable the effective loading of small molecule drugs and proteins,^[^ ^103^ ^]^ supporting slow and controlled release. However, current drug delivery routes using CaCO₃ nanoparticles primarily rely on traditional injection or oral administration. The most common method for preparing calcium carbonate nanomaterials is complexation, where water‐soluble calcium salts react with water‐soluble carbonates in solution to form calcium carbonate nanocarriers. Dong et al. developed monodisperse CaCO₃ nanoparticles modified with PEG as multifunctional nanocarriers.^[^ ^104^ ^]^ These nanoparticles were efficiently loaded with Mn^2^⁺‐chelated chlorin e6 (Ce6(Mn)) as a photosensitizer and doxorubicin (DOX) as a chemotherapeutic agent (**Figure** **6**A,(i,ii)). Following intravenous injection, the CaCO₃@Ce6(Mn)/DOX‐PEG nanoparticles progressively accumulated in tumors (Figure 6B,(i)), delivering superior synergistic antitumor effects by combining photodynamic and chemotherapeutic treatments (Figure 6B,(ii)).

**Figure 7:**
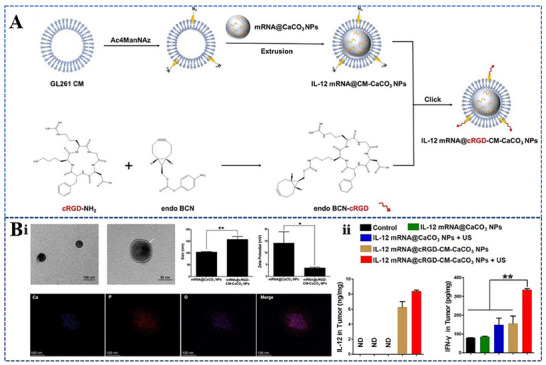
Preparation and characterization of mRNA@cRGD‐CM‐CaCO3 NPs. A) Illustration of the preparation of IL‐12 mRNA@cRGD‐CM‐CaCO3 NPs. B) Characterization of mRNA@cRGD‐CM‐CaCO3 NPs (i) and expression of IL‐12 and IFN‐γ in tumors of immunized mice(ii). Reproduced with permission.^[^ ^104^ ^]^ Copyright 2022 BioMed Central.

Although the current use of calcium carbonate nanomaterials as drug delivery carriers is limited to drug delivery, they also have the ability to deliver mRNA. Zhao et al. developed a novel biomimetic nanoparticle comprising a cRGD‐modified cytosolic shell and a calcium carbonate core containing IL‐12 mRNA (IL‐12mRNA@cRGD‐CM‐CaCO₃ NPs) (**Figure** **7**A).^[^ ^105^ ^]^ Polyacrylic acid‐stabilized biodegradable amorphous calcium carbonate nanoparticles have been employed for pH‐responsive drug delivery systems, significantly enhancing tumor suppression efficacy (Figure 7B). Liu et al. suggest that the strategy of preferential uptake by cancer cells through the inhalation of IL‐12 messenger RNA leads to targeted delivery and fewer systemic side effects.^[^ ^106^ ^]^ IL‐12 messenger RNA produces interferon‐gamma in innate and adaptive immune cell populations that can be activated to trigger a strong activation state and enhance immunogenicity in the tumor microenvironment. The increase in immune response leads to an expansion of tumor cytotoxicity in immune effector cells, immune memory formation, improved antigen presentation, and tumor‐specific T‐cell initiation.

Beyond small molecule chemotherapeutic drugs, amorphous calcium carbonate nanoparticles have also shown potential for gene therapy. They have been effectively used to deliver small interfering RNAs (siRNAs),^[^ ^107^ ^]^ with functionalized modifications enabling specific and highly efficient transfection of siRNAs into target cells. This success in siRNA delivery provides insights into the potential of calcium carbonate nanoparticles for mRNA delivery. Calcium carbonate nanomaterials demonstrate significant potential in drug delivery and cancer therapy owing to their pH sensitivity and slow‐release properties, which enhance therapeutic effects and minimize side effects. Multifunctional nanocarriers based on calcium carbonate nanoparticles offer a promising platform for combination therapies. To fully exploit their clinical potential, it is necessary to further refine the preparation methods to enhance their stability and increase drug‐loading capacity. Additionally, their applicability and effectiveness across different diseases must be further explored. Key areas for future research include investigating the metabolic pathways and potential in vivo toxicity of calcium carbonate nanoparticles to ensure safety. For mRNA delivery, exploring transfection efficiency and stability, along with comparative advantages over other delivery systems, is essential to establish calcium carbonate nanoparticles as a versatile and effective platform for therapeutic applications, which will provide a reference for the future delivery of mRNAs in treating lung diseases.

### 4.8 Exosomes

**Figure 8:**
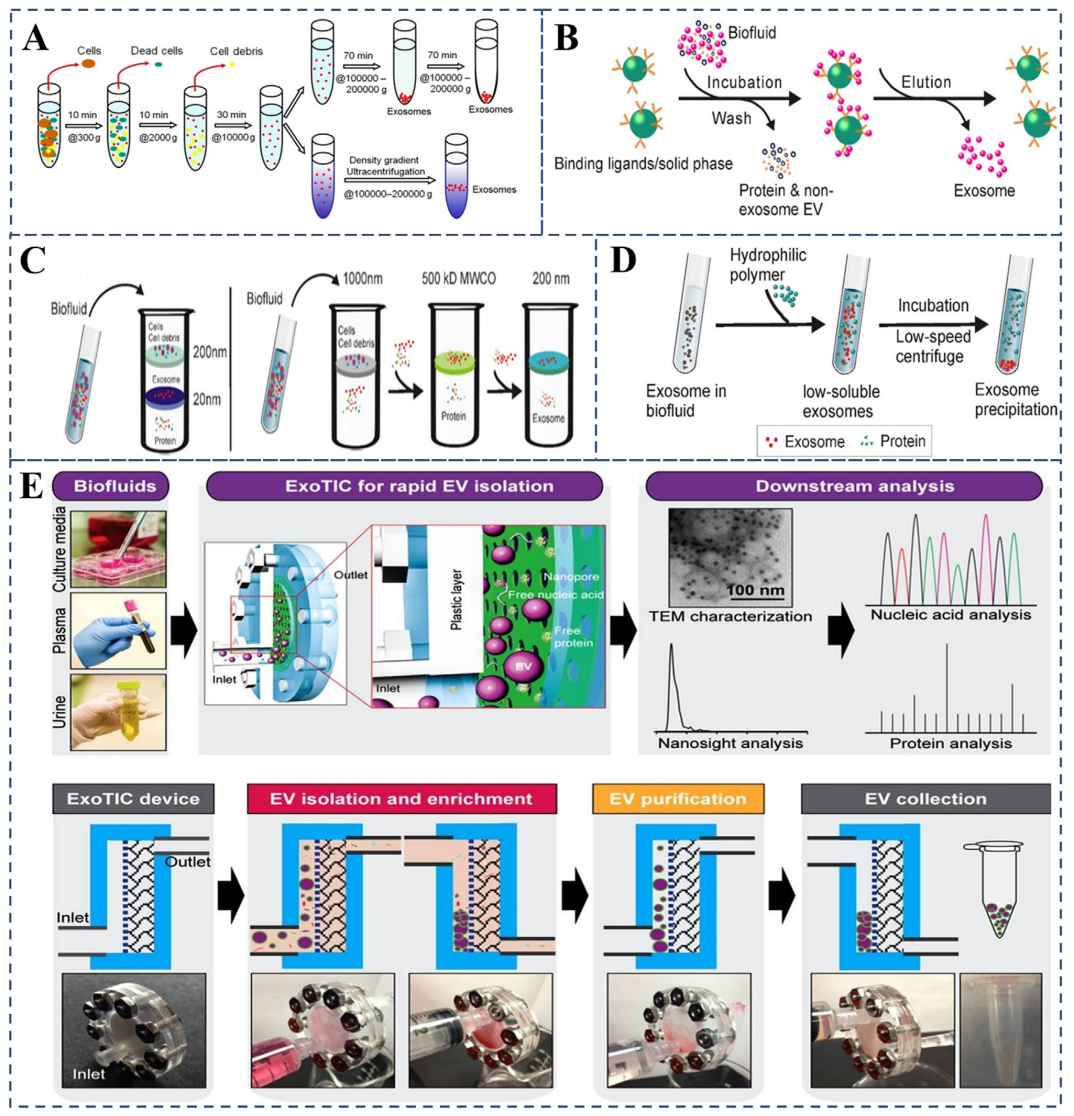
Exosomes were extracted by different methods. A) Schematic illustration of exosome isolation via differential ultracentrifugation, with all centrifugation steps conducted at 4 °C. Reproduced with permission.^[^ ^120^ ^]^ Copyright 2021 Pin Li. Ivyspring International. B) Schematic illustration of exosome isolation based on immunoaffinity. C) Schematic illustration of exosome separation via ultrafiltration. D) Schematic of polymer precipitation strategy for exosome isolation. Reproduced with permission.^[^ ^113^ ^]^ Copyright 2020 Ivyspring International. E) Schematic diagram of the ExoTIC system for the isolation of extracellular vesicles. Reproduced with permission.^[^ ^113^ ^]^ Copyright 2017 ACS.

Exosomes, which are also referred to as intraluminal vesicles (ILVs), are nanoscale membranous vesicles encapsulated within a single outer membrane.^[^ ^108^ ^]^ They enable the rapid, cost‐effective, and efficient isolation of nanoscale extracellular vesicles (nEVs) and the identification of genetic mutations in nEV, stem, and tumor cells, which can secrete exosomes.^[^ ^108^ ^]^ Exosomes are characterized by a lipid bilayer membrane structure akin to cell membranes, which protects the biomolecules contained within. These vesicles are rich in bioactive molecules, such as proteins, nucleic acids (e.g., mRNAs, miRNAs), and lipids, reflecting the physiological state of their originating cells.^[^ ^109^ ^]^ Exosomes are relatively stable in body fluids, enabling them to persist and function over extended periods. They can also be internalized by specific cells, facilitating the targeted delivery of bioactive molecules. This unique property renders exosomes an attractive platform for therapeutic and diagnostic applications. Exosomes are natural, nanoscale membrane vesicles with a lipid bilayer structure rich in bioactive molecules.^[^ ^110^ ^]^ They have a variety of specific molecules on their surface to interact with corresponding receptors on targeted cells for natural binging. The specificity and strength of this binding can be enhanced by genetically engineering the exosome‐producing parental cells to express specific surface molecules.^[^ ^111^ ^]^ Various methods have been used to isolate exosomes, including differential ultracentrifugation^[^ ^112^ ^]^ (**Figure** **8**A), ultrafiltration^[^ ^102^ ^]^ (Figure 8B), immunoaffinity capture^[^ ^113^ ^]^ (Figure 8C), and sedimentation^[^ ^114^ ^]^ (Figure 8D). Emerging technologies, such as microfluidics^[^ ^115^ ^]^ (Figure 8E) and innovative sorting mechanisms such as acoustic,^[^ ^116^ ^]^ electrophoretic,^[^ ^117^ ^]^ and electromagnetic^[^ ^118^ ^]^ manipulations, are also being explored. A novel lipid nanoprobe method has been developed that enables the rapid, cost‐effective, and efficient isolation of nEVs and the identification of genetic mutations in nEVs isolated from patients with non‐small cell lung cancer (NSCLC).^[^ ^119^ ^]^

Biologically derived exosomes offer excellent biocompatibility and minimal immune response activation. They effectively protect mRNA from nuclease degradation, enhancing its stability. Additionally, surface‐specific molecules on exosomes enable precise interactions with target cells, facilitating efficient targeted delivery.^[^ ^121^ ^]^ Their capacity to traverse biological barriers, including the blood–brain barrier^[^ ^122^ ^]^ and the pulmonary tissue barrier,^[^ ^123^ ^]^ facilitates targeted mRNA delivery to specific tissues and cells. Compared with synthetic delivery vehicles, exosomes are less immunogenic, minimizing the risk of immune rejection. Moreover, exosomes can simultaneously deliver multiple bioactive molecules, including mRNAs, siRNAs, miRNAs, and proteins, making them a versatile platform for combination therapies.^[^ ^124^ ^]^ Exosomes have shown significant therapeutic potential in various lung diseases. For instance, in COPD, exosomes can carry anti‐inflammatory and antioxidant molecules, such as miRNAs, to lung tissues, reducing inflammation and oxidative stress. Tan et al. found elevated circulating exosomes in both stable COPD and exacerbation phases, correlating with systemic inflammatory biomarkers.^[^ ^125^ ^]^ Similarly, in asthma, exosomes deliver miRNAs that modulate immune cell function to suppress inflammation and specific mRNAs that improve the contractile function of airway smooth muscle cells, alleviating the symptoms.^[^ ^126^ ^]^ For pulmonary fibrosis, exosomes carrying anti‐fibrotic molecules, such as TGF‐β inhibitors, can inhibit disease progression by promoting lung fibroblast apoptosis and reducing collagen deposition.^[^ ^127^ ^]^ In lung cancer, exosomes serve as drug carriers, delivering chemotherapeutic and immunotherapeutic agents directly to tumor cells to enhance treatment efficacy. Exosomes carrying tumor‐specific antigens can activate the immune system to generate antitumor responses.^[^ ^128^, ^129^ ^]^ Surface modification of exosomes can significantly improve their stability, targeting ability, and biocompatibility. Genetic modification of exosome‐producing parent cells facilitates the expression of specific targeting molecules, thereby enabling the precise delivery of exosomes to particular cells or tissues.^[^ ^130^ ^]^ For example, in the treatment of lung cancer, anti‐epidermal growth factor receptor (EGFR) antibodies can be conjugated to exosomes to specifically target cancer cells that overexpress EGFR.^[^ ^131^ ^]^ However, challenges remain, including the potential impact of surface modifications on exosome stability and biological activity. The safety and effectiveness of different modification methods must be evaluated to ensure their clinical feasibility. While exosomes exhibit exceptional biocompatibility, their isolation and purification processes remain inherently complex and yield low quantities. Current techniques—such as differential ultracentrifugation and immunoaffinity capture—are labor‐intensive, require specialized equipment, and are not well‐suited for large‐scale production. For instance, isolating exosomes from stem cell or tumor cell cultures typically yields only microgram quantities per liter of culture, significantly limiting therapeutic scalability.^[^ ^132^ ^]^  Furthermore, batch‐to‐batch variability in exosome size, cargo composition, and purity poses additional challenges for standardized manufacturing and clinical translation.

Isolation and purification of exosomes also require optimization to improve yield and purity. We summarized the advantages and disadvantages of biomaterials in terms of delivery mode, mRNA loading capacity, transfection efficiency, and lung residence time, as shown in **Table** **1**.

**Table 1:** Comparison of different biomaterials in terms of delivery mode, mRNA loading capacity, transfection efficiency, and lung residence time.

Biomaterials	Delivery method	mRNA loading capacity	Transfection efficiency (lung)	Lung retention time	advantages	disadvantages	Refs.
**Liposomes**	Nebulized Inhalation, Intravenous	5%–15%	Alveolar epithelial cells Greater than 80%, lung endothelial cells > 70%.	24–48 h	1. Good biocompatibility and targeting. 2. Protects mRNA from degradation. 3. Easy surface modification (e.g., PEGylation, antibody coupling). 4. Lung targeting by nebulized inhalation.	1. Cationic lipids may trigger cytotoxicity and immune reactions. 2. Less stable and susceptible to lung surfactants. 3. Complex and costly preparation process.	[38, 57, 133, 134]
**Polymer Nanoparticles**	Nebulized inhalation	3%–10%	Bronchial epithelial cells 60–70%, lung endothelial transfection rate 50–60%	12–24 h	1. Enhances cellular uptake by modulating charge and size. 2. Biodegradable and low toxicity. 3. Supports slow release and pH responsive release. 4. Suitable for nebulized inhalation.	1. Lower transfection efficiency than liposomes. 2. Poor water solubility of natural polymers (e.g., chitosan). 3. Long‐term in vivo metabolism mechanism is unclear.	[73, 74, 135, 136]
**Lipid–polymer** **Hybrid Nanoparticles**	Nebulized inhalation, intravenous	10%–20%	Alveolar macrophages Greater than 70%	48–72 h	1. Combines the advantages of liposomes and polymers, high stability. 2. Can load hydrophobic/hydrophilic drugs simultaneously. 3. Flexible surface modification, strong targeting.	1. Complex preparation process, need to precisely control the proportion of components. 2. In vivo distribution and toxicity need to be further verified.	[81, 82, 86]
**Dendritic Polymers**	Intratracheal injection	8%–15%	Lung fibroblasts 50–60%	12–36 h	1. Highly branched structure with high drug‐carrying capacity. 2. Rich surface functional groups, easy to be modified. 3. Efficient delivery through endocytosis.	1. High charge density may lead to cytotoxicity. 2. Fast clearance rate in vivo, PEGylation required to improve circulation time.	[87, 90]
**Gold Nanoparticles**	Nebulized inhalation	5%–8%	Tumor cells 40–50%	24–48 h	1. Photothermal effects can be combined (e.g., photothermal and gene therapy). 2. Biocompatible and easy to surface functionalize. 3. Can be used for dual‐modality imaging (CT/PAI)	1. High cost, difficult to produce on a large scale. 2. Long‐term accumulation in the body may cause toxicity.	[94, 137, 138]
**Mesoporous Silica Nanoparticles**	Intratracheal injection	15%–25%	Airway epithelial cells greater than 90%	More than 72 h	1. High surface area and porosity, high drug‐carrying capacity. 2. High chemical stability, resistant to enzymatic degradation. 3. Surface modification of targeted groups.	1. Inorganic materials may cause inflammatory reactions. 2. Poor degradability in vivo, long‐term safety concerns.	[98, 99]
**Calcium Carbonate Nanomaterials**	Nebulized inhalation	3%–6%	Immune cells 30–40%	6–12 h	1. pH‐responsive release, targeting acidic. microenvironments. 2. Biocompatible, slow degradation. 3.Simple preparation, low cost.	1. Low drug loading efficiency, dependent on physical adsorption. 2. Uneven particle size distribution may affect delivery efficiency.	[104, 105]
**Exosomes**	Intravenous injection	1%–3%	Systemic distribution, lungs enriched 20–30	6‐24 h	1. Naturally biocompatible, low immunogenicity. 2. Crosses biological barriers (e.g., blood–lung barrier). 3. Endogenous targeting capabilities (e.g., stem cell‐derived exosomes).	1. Difficult to isolate and purify, low yield. 2. Limited drug‐carrying modes (electroporation or co‐culture required). 3.Poor stability, cold chain storage required.	[124, 125, 126, 127]

## 5 Biomaterial Delivery of mRNA in the Treatment of Lung Diseases

According to the latest statistics, the total number of patients with respiratory diseases in China has exceeded 100 million. Among them, there are close to 100 million patients with COPD over the age of 20, ≈50 million asthma patients aged 20 and above, and ≈8.7 million asthma patients among children and adolescents. In addition, there are ≈781 000 new cases of lung cancer each year. At the same time, the drug delivery technology of biomaterials in the treatment of lung diseases is constantly being innovated and gradually improved. We summarized the biomaterials for treating different lung diseases, as shown in **Table** **2**.

**Table 2:** Biomaterials in the treatment of different lung diseases.

Disease	Name	Strategy	Strengths	Refs.
Pulmonary Fibrosis	Ribosomal protein‐concentrated mRNA	Nanopreparations consisting of mRNA cores concentrated from ribosomal proteins	Enhanced expression of matrix metallopeptidase 13 ameliorates pulmonary fibrosis	[142]
	LNP‐based mRNA therapy	LNP provides efficient mRNA encapsulation	Effectively prevents mRNA degradation and sustains release over time	[78]
	Embedding of IL‐11scFv mRNA in iLNP	Can rapidly escape from lysosomes, release mRNA into the cytoplasm, and consistently express IL‐11scFv	Inhaled scFv@iLNP‐HP0 also significantly enhances lung function recovery in bleomycin‐induced fibrotic mice	[143]
Asthma	Foxp3mRNA Tlr1/2mRNA, Tlr2/6mRNA	High‐pressure endotracheal spray delivery to the lungs by optimizing modified mRNAs	Efficient, reproducible, site‐specific, and naturally self‐limiting upregulation of Foxp3	[186]
	Increasing LNP charge during atomization	Tlr1/2mRNA or Tlr2/6mRNA co‐treatment	can improve lung function and reduce airway inflammation in the body	[145]
		LNP formulations can be stabilized against atomization‐induced aggregation by the addition of branched polymer excipients	Significant improvement in pulmonary mRNA delivery	[146]
Pneumonia	8‐9DmRNA LNP PBAE formulation p53 mRNA Nanoparticles	Optimizing the formulation of LNPs, mRNA sequences of antibodies, and applying cyclic RNA technology	Achieves lung‐selective delivery of 8–9D mRNA, a broadly neutralizing antibody against SARS‐CoV‐2; improves mRNA stability and expression	[154]
		A poly beta‐amino thioester (PBATE) that efficiently delivers mRNA to mice	Produces similar efficacy to neutralizing antibodies at 20‐fold higher doses	[135]
Lung		Dual‐targeted mRNA nano‐formulations using novel cationic lipid and hyaluronic acid bases	Demonstrates anticancer effects on	[162]
Cancer	LNP‐CAD9	Combinatorial synthesis of 180 cationic, degradable lipids, in vitro screening combined with in vivo barcoding technology	H1299 lung cancer cells in in vitro experiments Lungs have the highest mRNA delivery efficiency; tumor growth significantly inhibited by CRISPR‐Cas9 gene editing in a lung cancer model retention time of COPD drugs in the lungs	[163]
Chronic Obstructive	Amphiphilic PBAE nanoparticles	Endothelial‐specific targeting and efficient delivery in the lung by altering the ratio of the two alkyl chains in the backbone	LNP endocytosis improves local concentration and	[173, 174]
Chronic Obstructive Pulmonary Disease (COPD)	Polyethylene glycolyzed liposomes	Altering the surface of LNPs helps them overcome the clearance mechanism	Remains in the airway longer and releases its payload over a more sustained period, resulting in increased localized drug concentration and therapeutic effect	[176, 187]
Acute Respiratory	DOTAP‐LNPs and sPD‐L1 mRNAs	Addition of lipid nanoparticle (LNP) delivery using DOTAP	Delivers soluble programmed death ligand PD‐L1 (sPD‐L1). mRNA can be specifically expressed by lung tissue, leading to in situ immunosuppression in ARDS lung tissue	[178]
Distress Syndrome (ARDS)	Green fluorescent protein IκBα‐SR or SOD3 mRNA	The mRNA encoding the green fluorescent protein IκBα‐SR or SOD3 was complexed with cationic lipids, passed through a vibrating mesh nebulizer, and delivered to cell cultures or directly to rats with E. coli pneumonia	SOD3mRNA improves static lung compliance and alveolar‐arterial oxygen gradient (AaDO2) and reduces bacterial load in bronchoalveolar lavage (BAL) fluid	[182]

### 5.1 Treatment of Pulmonary Fibrosis

Pulmonary fibrosis (PF) is a chronic, progressive interstitial lung disease characterized by a distinctive histopathological pattern, encompassing various chronic respiratory conditions marked by the growth of connective tissue in lung cavities. Among these conditions, interstitial lung disease and idiopathic pulmonary fibrosis (IPF) are the most severe and irreversible forms, leading to progressive lung parenchymal fibrosis. PF is characterized by abnormal lung tissue repair and excessive accumulation of extracellular matrix. These pathological processes lead to high morbidity and mortality rates, ultimately causing loss of lung function and death.^[^ ^139^ ^]^ In 2014, the U.S. Food and Drug Administration (FDA) approved two medications—pirfenidone and nintedanib^[^ ^140^ ^]^—to manage PF. Clinical studies have shown that these drugs can delay the deterioration of lung function and the progression of IPF. However, current therapeutic options are limited to delaying disease progression and are unable to achieve a complete cure. Additionally, these treatments are linked to adverse side effects, including gastrointestinal bleeding and severe diarrhea, with no clear evidence of improvement in long‐term mortality.

**Figure 9:**
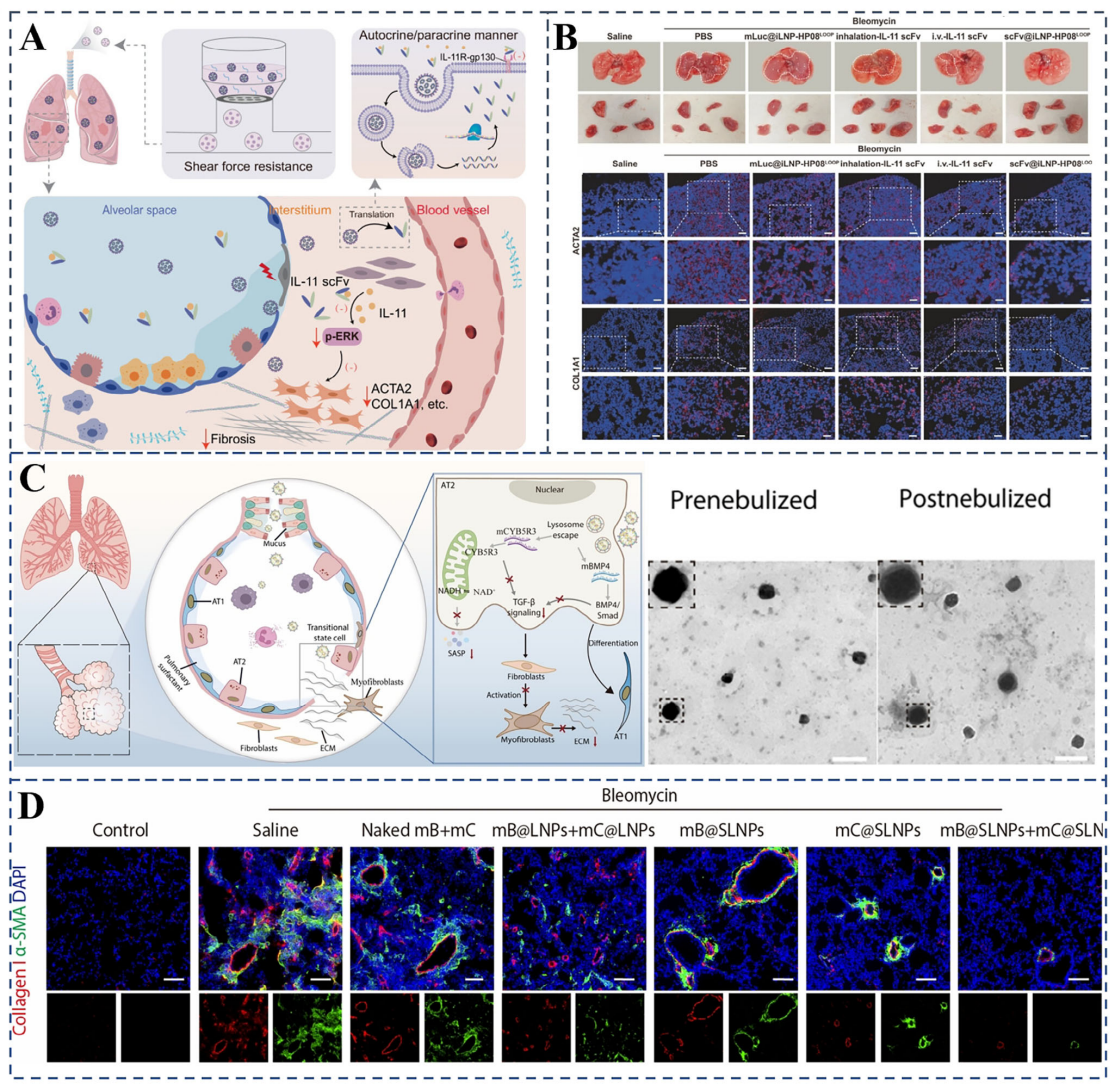
scFv@iLNP‐HP08LOOP for the treatment of IPF. A) Inhaled delivery of scFv@iLNP‐HP08LOOP for the treatment of IPF. B) Representative lung tissues from mice treated with saline, bleomycin + inhaled PBS, bleomycin + inhaled mLuc@iLNP‐HP08LOOP, bleomycin + inhaled IL‐11 scFv, bleomycin + intravenous IL‐11 scFv, and bleomycin + inhaled scFv@iLNP‐HP08LOOP, as well as COL1A1 and ACTA2 immunofluorescence images and quantitative analysis of ACTA2. Reproduced with permission.^[^ ^134^ ^]^ Copyright 2024 Springer. C) Schematic and characterization of inhaled delivery of mRNA‐LNP to AT2 cells to reverse epithelial stem cell depletion to treat IPF. D) Representative images of lung alpha‐SMA (green) and collagen I (red) immunostaining after different group treatments. Reproduced with permission.^[^ ^144^ ^]^ Copyright 2024 AAAS.

Despite the intricate and not fully elucidated nature of PF pathogenesis, research indicates that oxidative stress is a pivotal factor. This stress is primarily initiated by continuous damage to type II alveolar epithelial cells (AECsII) and is further exacerbated by the diminished expression of nuclear factor erythroid 2‐related factor 2 (Nrf2).^[^ ^141^, ^142^ ^]^ Zhang et al.^[^ ^143^ ^]^ revealed the therapeutic potential of a nano‐formulation containing ribosomal proteins condensed with mRNA cores to enhance matrix metallopeptidase 13 (MMP13) expression, which improved disease progression in a bleomycin‐induced mouse model of PF. Massaro et al. emphasized the potential of LNP‐mediated mRNA therapies for effectively delivering mRNA to fibrotic lung tissues.^[^ ^78^ ^]^ Their findings showed that iLNP efficiently encapsulated mRNA, prevented degradation, and provided sustained release over time. Recently, Bai et al.^[^ ^134^ ^]^ demonstrated that iLNP encapsulating IL‐11scFv mRNA efficiently entered cells, escaped lysosomes, released mRNA into the cytoplasm, and sustained IL‐11scFv expression (**Figure** **9**A). Histological analysis demonstrated that treatment with scFv@iLNP‐HP08 effectively mitigated the thickening of alveolar septa induced by bleomycin, repaired damaged alveolar structures, decreased collagen accumulation, and parenchymal damage. Inhalation of scFv@iLNP‐HP08 resulted in a marked improvement in lung function in fibrotic mice, as demonstrated by hematoxylin and eosin (H&E) staining and immunofluorescence analysis. These results highlight the therapeutic potential of LNP‐based mRNA delivery for managing IPF (Figure 9B). Although all tested strategies achieved mitigation of PF, their mechanisms varied. Zhang enhanced protein expression by delivering MMP13 via intravenous ribosomal protein nanoparticles. Massaro et al. achieved sustained suppression of fibroblast activation through inhaled mRNA therapy. Bai, in contrast, targeted the IL‐11/IL‐11R signaling pathway by delivering IL‐11 scFv using scFv@iLNP via inhalation. Among these approaches, targeting the IL‐11R pathway demonstrated the greatest therapeutic efficacy. The advancement of LNP‐based mRNA therapeutics offers new hope for treating PF by enabling the targeted enhancement of specific gene expression. However, these studies are in their early stages, and further studies are required to assess their safety and effectiveness. Additionally, a more comprehensive grasp of the mechanisms underpinning mRNA therapy is crucial for refining therapeutic approaches and promoting clinical advancements. Wang et al. designed an inhalable mucus‐penetrating lipid nanoparticle (LNP) for co‐delivery of dual mRNA to promote alveolization for IPF treatment by restoring AT2 stemness (Figure 9C).^[^ ^144^ ^]^ Upon inhalation in a bleomycin model, LNP reversed mitochondrial dysfunction by improving nicotinamide adenine dinucleotide biosynthesis, thereby inhibiting accelerated senescence of AT2 (Figure 9D).

### 5.2 Treatment of Asthma

**Figure 10:**
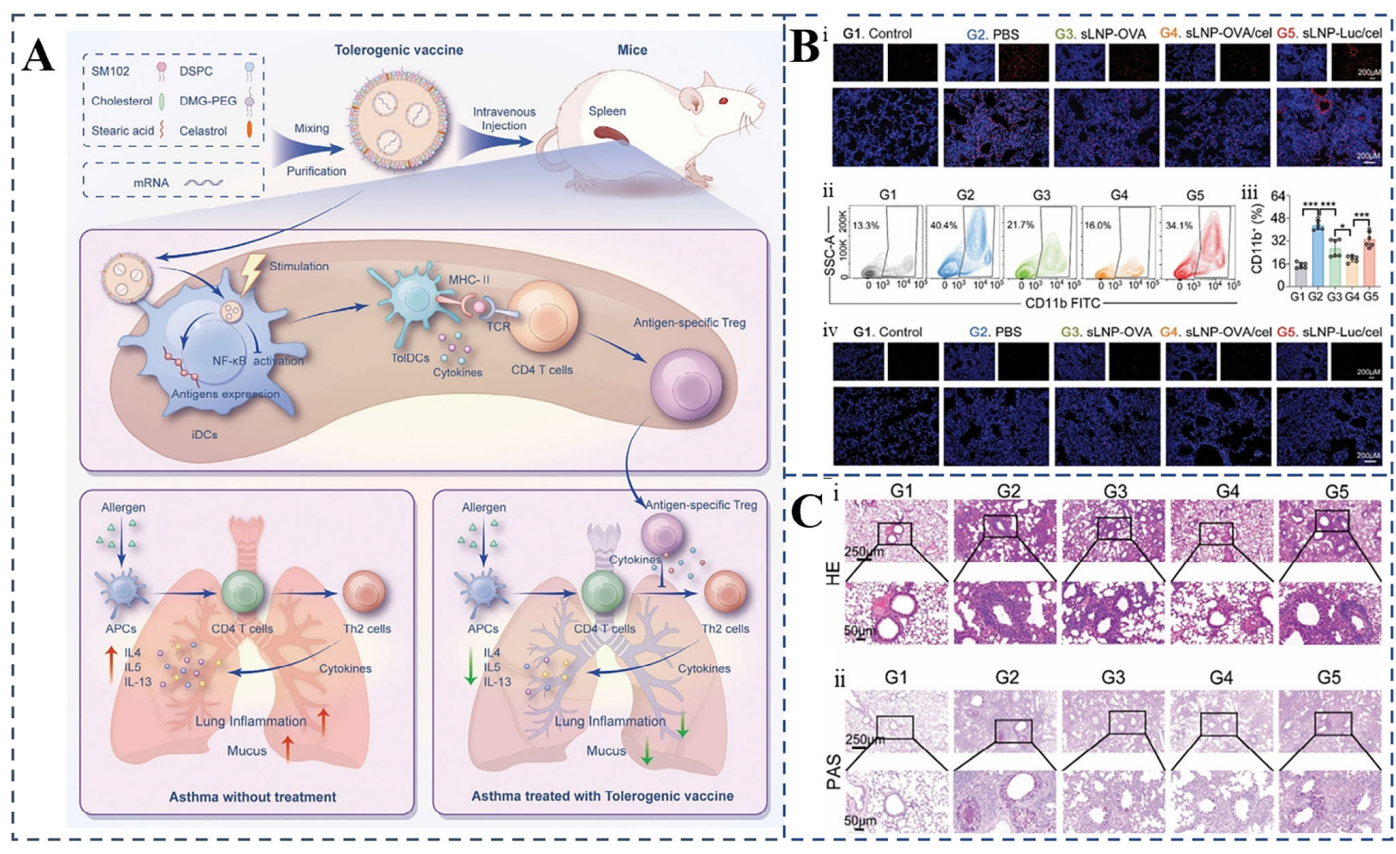
mRNA‐LNPs treatment of asthma. A) Schematic diagram of spleen dendritic cells targeting experimental asthma tolerance mRNA‐LNPs vaccine. Experimental allergic asthma was treated by inducing tolerance of dendritic cells and subsequent antigen‐specific CD4+ regulatory T cells (Treg) mediated immune tolerance, targeting nucleoside‐modified antigen mRNA and CEL‐supported LNPs vaccine. B) Immunomodulatory effects of sLNP‐OVA/Cel on the lungs of asthmatic mice. Representative immunofluorescent staining images of anti‐CD11b antibody lungs from asthmatic mice treated with PBS and nanovaccines(i). Quantitative analysis of inflammatory monocyte infiltration in the lungs by flow cytometry (ii, iii). Representative immunofluorescence staining images of lungs with anti‐FOXP3 antibodies from control and nanofaxine‐treated asthmatic mice (iv). C) Representative H&E staining images of asthmatic mouse lungs treated with PBS and nanovaccines (i). Representative PAS staining images of asthmatic mice lungs treated with control and nanovaccines at the end of the study(ii). Reproduced with permission.^[^ ^148^ ^]^ Copyright 2025 Wiley.

Although no definitive cure exists for asthma, emerging research explores the potential of modified mRNAs to deliver therapeutics directly to target sites, offering preventive or therapeutic benefits. Mays et al. designed a novel IL‐10‐mediated treatment for allergic asthma using modified Foxp3 mRNA.^[^ ^145^ ^]^ This method enabled efficient, targeted, and self‐regulating enhancement of Foxp3 protein expression in the lungs by delivering the modified mRNA using a high‐pressure intratracheal spray technique. The findings indicate that Foxp3 mRNA may be a valuable target for developing preventive and therapeutic approaches in allergic asthma and related conditions. Similarly, Zeyer et al. demonstrated that treatment with Tlr1/2 mRNA or Tlr2/6 mRNA, but not Tlr2 mRNA alone, improved lung function and reduced airway inflammation in vivo.^[^ ^146^ ^]^ The results indicate that TLR heterodimers may exert a protective effect in the pathogenesis of asthma. Jiang et al. further advanced lung mRNA delivery techniques by modifying the nebulization buffer to enhance LNP charge during nebulization and by adding a branched‐chain polymer excipient to stabilize LNPs against nebulization‐induced aggregation.^[^ ^136^ ^]^ This combination significantly enhanced lung mRNA delivery efficiency. Although mRNA‐based strategies show promise in improving therapeutic outcomes through efficient targeting, these approaches are still in the laboratory stage. Further clinical studies are required to validate their safety and efficacy. Additionally, given the individual variability among patients, developing personalized treatment plans to optimize therapeutic success is crucial. Recently Wang et al. demonstrated that stearic acid‐doped LNP co‐loaded with nucleoside‐modified mRNA and celastrol selectively targeted the spleen, transformed its adjuvant, and promoted tolerogenic rather than immunogenic DCs phenotype.^[^ ^147^ ^]^ In addition, the tolerogenic mRNA vaccine promotes the generation of antigen‐specific regulatory T cells (Tregs) in the spleen and facilitates the migration of induced Tregs to the lungs (**Figure** **10**A). In a mouse model of allergic asthma, immunization with the tolerogenic mRNA vaccine significantly alleviated symptom onset, reduced eosinophil infiltration, and decreased mucus secretion. The nanovaccine‐treated group exhibited a notable reduction in inflammatory monocyte infiltration in the lungs compared to the PBS control group, confirming both the attenuation of pulmonary inflammation and the corresponding increase in FOXP3‐expressing regulatory T cells, thereby indicating an enhanced immunosuppressive microenvironment (Figure 10B,(i–iv)). Furthermore, H&E staining (i) and periodic acid–Schiff (PAS) staining (ii) revealed a significant reduction in tissue structural damage, contributing to improved asthma symptoms in treated mice (Figure 10C,(i,ii)).

### 5.3 Treatment of Pneumonia

Viruses, including the novel coronavirus (SARS‐CoV‐2), present complex challenges in treatment due to their ability to mutate and produce new strains, such as Delta and Omicron. These mutations often alter characteristics such as infectivity and pathogenicity, complicating prevention, control, and treatment efforts. Although antiviral^[^ ^149^, ^150^, ^151^, ^152^ ^]^ and anti‐inflammatory drugs^[^ ^153^, ^154^ ^]^ have been used to treat COVID‐19 pneumonia, no specific antiviral drug for SARS‐CoV‐2 exists. Drug development and validation require significant time; moreover, individual responses to available treatments widely vary. Additionally, COVID‐19 pneumonia often involves multiple organ systems, such as the heart, kidneys, and liver, further complicating diagnosis and treatment. Conventional drugs primarily provide symptomatic relief and are associated with low drug utilization efficiency. In contrast, the effectiveness of vaccines for the treatment of pneumonia has been proven.^[^ ^155^ ^]^

**Figure 11:**
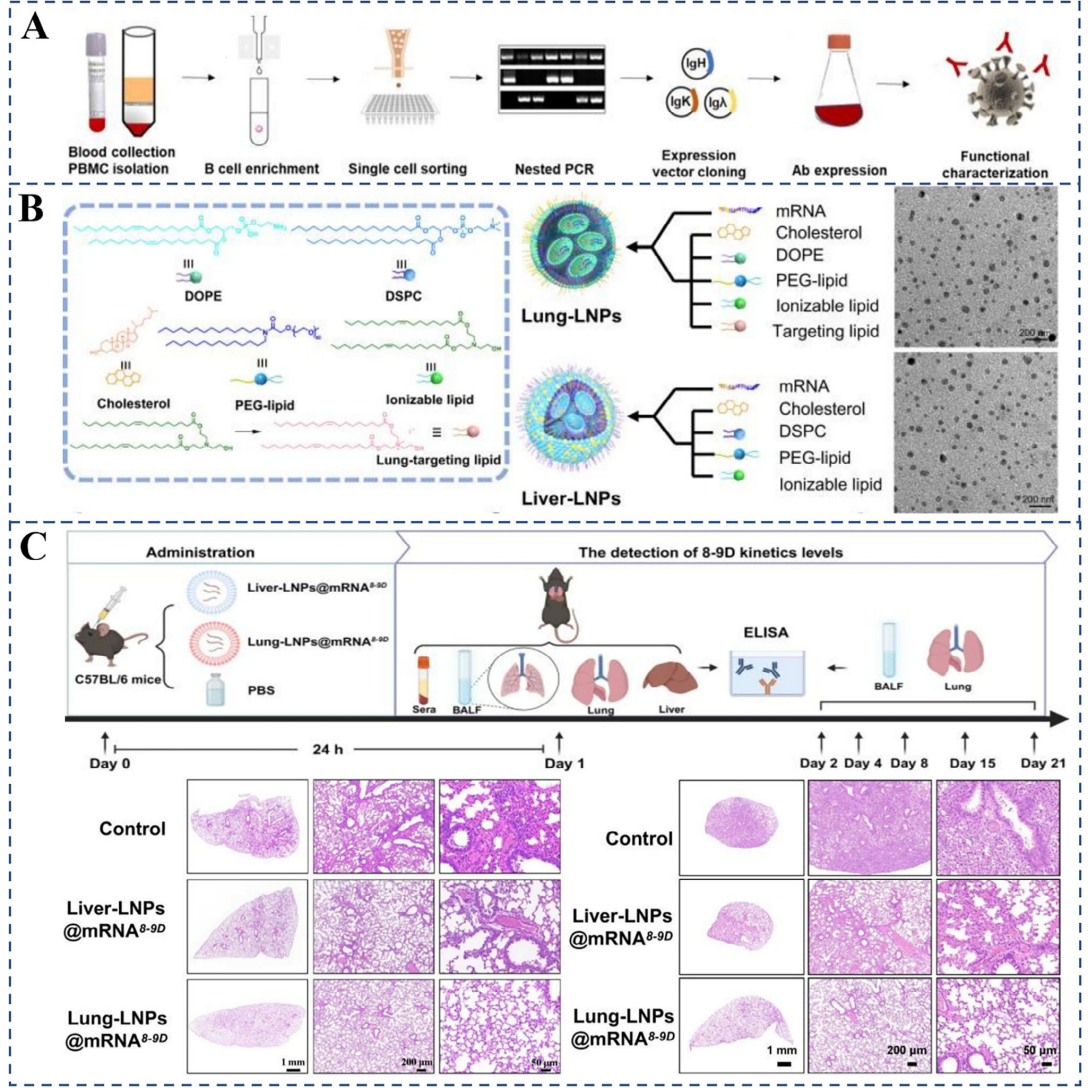
Monoclonal antibody 8–9D targeting SARS‐CoV‐2. A) Schematic depiction of the process for selecting and evaluating neutralizing monoclonal antibodies. B) Characterization of organ‐selective lipid nanoparticle systems. C) K18‐hACE2 mice were injected intravenously with 5 µg liver LNPs@mRNA8‐9D or lung LNPs@mRNA8‐9D, and the lungs were immobilized for histological evaluation. Reproduced with permission.^[^ ^157^ ^]^ Copyright 2023 Springer.

Emerging therapies leveraging mRNA technology offer promising solutions for pneumonia treatment. Studies have shown that nebulized delivery of mRNA‐encoded neutralizing monoclonal antibodies (mAbs) as a prophylactic polymer significantly decreased viral titers, viral RNA levels, lung pathology, and infection‐induced body weight loss in a hamster model of SARS‐CoV‐2.^[^ ^156^ ^]^ Tai et al. identified a human monoclonal antibody 8–9D (**Figure** **11**A) that demonstrated strong neutralization against multiple SARS‐CoV‐2 variants by modifying LNP (Figure 11B).^[^ ^157^ ^]^ Using a lung‐selective mRNA delivery platform, 8–9D mRNA expression in the lungs effectively prevented viral invasion, protecting female K18‐hACE2 transgenic mice from Beta and Omicron BA.1 variants (Figure 11C). Rotolo et al. advanced pulmonary mRNA delivery by developing a poly‐β‐aminothioester (PBATE) formulation, P76, using a combinatorial synthetic strategy and a low dead‐volume nebulizer‐based particle screening system.^[^ ^135^ ^]^ P76 demonstrated efficient mRNA delivery across diverse species, including mice, hamsters, ferrets, cows, and rhesus monkeys, regardless of RNA cargo size and complexity. It exhibited high safety and tolerability, with greater expression than prior aerosolized poly‐β‐aminoester (PBAE) candidates. In a Syrian hamster model targeting SARS‐CoV‐2, P76 delivered Cas13a‐mediated therapy with a four‐fold dose savings compared with earlier nebulized PBAE formulations. Remarkably, the therapy achieved similar efficacy as a neutralizing antibody administered at a 20‐fold higher dose. The combinatorial synthesis approach enables the development of advanced polymeric formulations for liposomal mRNA delivery, opening new possibilities for RNA‐based drug development in pneumonia treatment. However, to optimize therapeutic outcomes, it is crucial to consider the unique features of viral variants, enhance delivery systems, and improve the adaptability, stability, and targeting of mRNA to ensure effective expression in the lungs. These advancements offer significant potential for combating pneumonia and other viral respiratory diseases.

### 5.4 Treatment of Lung Cancer

Globally, lung cancer ranks as the second most prevalent cancer and is the primary cause of cancer‐related mortality. Lung cancer can be broadly categorized into two main types: small‐cell lung cancer and NSCLC. Among these, NSCLC is the most common form. Lung cancer treatment depends on the stage, tumor subtype, and the patient's overall health and comorbidities. Standard therapies include surgery, radiotherapy, and chemotherapy. Surgery remains the treatment of choice for early‐stage NSCLC, offering the highest chance of cure.^[^ ^158^ ^]^ In patients with early‐stage NSCLC who are not candidates for surgical resection, stereotactic ablative radiotherapy (SABR) serves as a viable alternative treatment option. SABR delivers high doses of radiation to the tumor using multiple narrow beams while minimizing radiation exposure to surrounding healthy tissue, reducing collateral damage. Chemotherapy, despite its significant adverse effects, remains a vital component in the treatment of lung cancer, both as a primary and supportive therapy. However, traditional intravenous chemotherapy necessitates the administration of high doses of cytotoxic agents, which can inflict significant damage on healthy tissues and organs, leading to severe adverse effects. mRNA‐based strategies have emerged as a promising alternative, offering precision and complementary therapeutic benefits—particularly when integrating immune checkpoint modulation with neoantigen delivery—to effectively counter tumor immune evasion.

**Figure 12:**
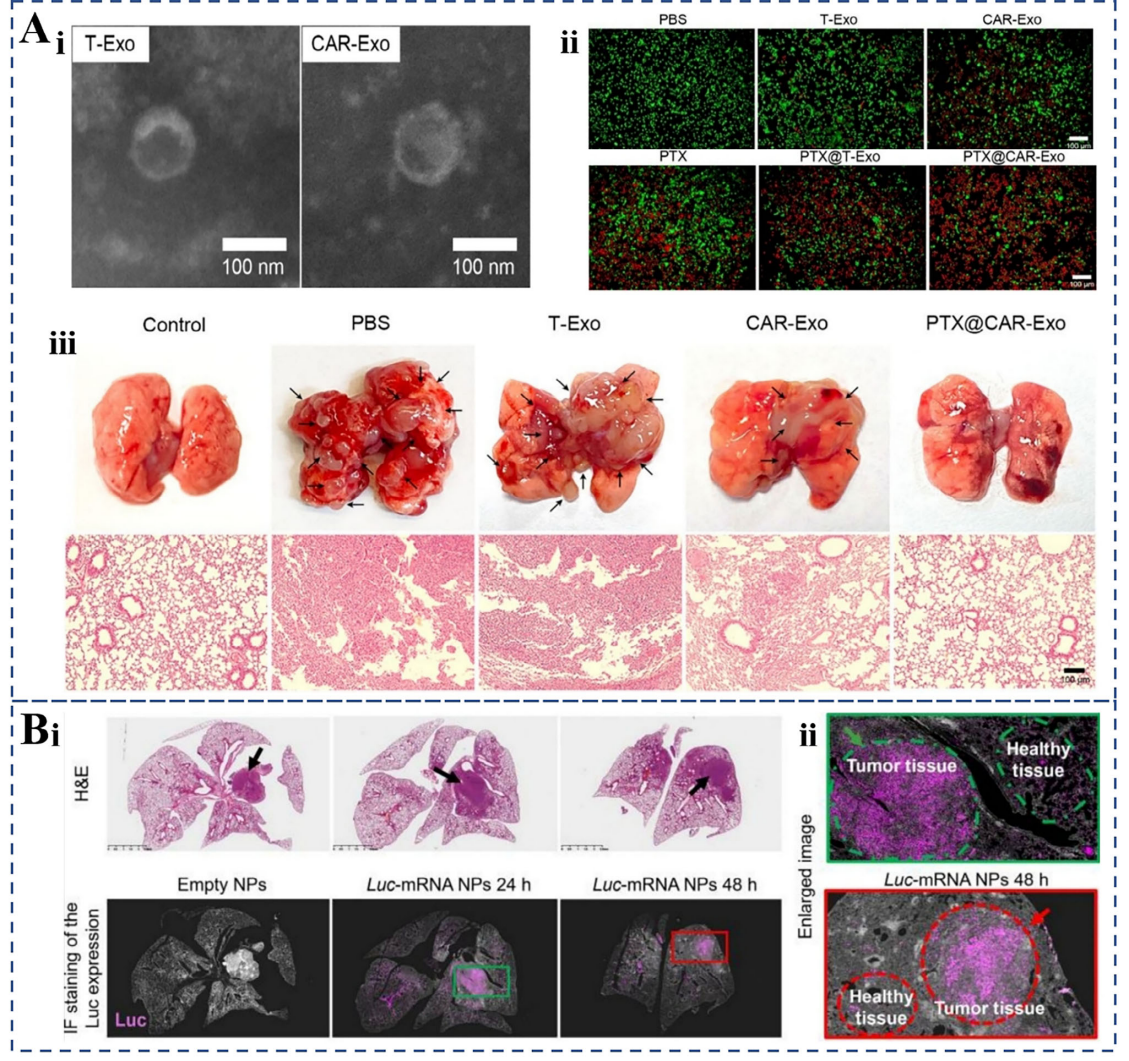
Inhalation therapy for lung cancer. A) Characterization(i) and cytotoxicity of CAR‐Exos and its therapeutic effect on tumors(ii, iii). Reproduced with permission.^[^ ^162^ ^]^ Copyright 2023 BioMed Central. B) Histological examination (H&E staining) of lung tissue in mice(i) with in situ non‐small cell lung cancer at IH at 24 and 48 h with empty HDPM NPs or Luc‐mRNA HDPM NPs(ii). Reproduced with permission.^[^ ^163^ ^]^ Copyright 2023 Proc Natl Acad Sci USA.

Localized drug delivery offers a promising alternative, as anti‐cancer drugs and small interfering RNAs (siRNAs) can be loaded into lipid carriers to specifically target lung cancer cells. For instance, Taratula et al. developed NLCS to deliver anticancer drugs and siRNAs through inhalation.^[^ ^58^ ^]^ This method facilitated precise delivery to tumors, effectively suppressing tumor growth and minimizing the impact on healthy tissues. Chemotherapeutic agents such as paclitaxel (PTX) remain central to lung cancer treatment. PTX inhibits tumor proliferation and modulates immune cells within the tumor microenvironment (TME), stimulating the release of cytotoxic molecules and tumor suppressor cytokines to enhance tumor cell elimination.^[^ ^159^, ^160^, ^161^ ^]^ Zheng et al. advanced this approach by designing chimeric antigen receptor (CAR)‐derived exosomes (CAR‐Exos) from T cells to deliver PTX to localized tumors.^[^ ^162^ ^]^ Administered through inhalation, CAR‐Exos achieved targeted delivery to lung tumors in mice, enhancing therapeutic efficacy and reducing adverse reactions (**Figure** **12**A,(i–iii)). This strategy overcame the limitations of CAR‐T cell therapy for solid tumors, such as cell depletion, while providing a novel delivery mechanism for chemotherapeutic agents. Tang et al. developed a dual‐targeted mRNA nano‐formulation using cationic lipid and hyaluronic acid modifications.^[^ ^163^ ^]^ This formulation showed significant anticancer activity against H1299 lung cancer cells in vitro by activating apoptotic signaling pathways through p53 protein expression, thereby inducing cancer cell apoptosis. This approach offers a promising strategy for treating p53 function‐dependent tumors (Figure 12B,(i,ii)). Additionally, Xue et al. synthesized a variety of cationic, degradable lipids (CAD lipids) to create an efficient mRNA delivery system.^[^ ^164^ ^]^ Using in vitro screening and in vivo barcoding techniques, they identified the LNP‐CAD9 formulation, which excelled in lung mRNA delivery. In a mouse model of lung cancer, LNP‐CAD9 significantly inhibited tumor growth and prolonged survival by utilizing CRISPR‐Cas9 technology.^[^ ^165^ ^]^

Lung cancer treatment frequently targets tumor‐associated antigens (TAAs) such as carcinoembryonic antigen (CEA) and EGFR. However, because TAAs are also expressed in normal tissues, their therapeutic utility is limited by reduced antitumor specificity. Additionally, the tumor microenvironment (TME) is inherently immunosuppressive, characterized by PD‐L1 overexpression, M2 macrophage polarization, and regulatory T cell (Treg) infiltration, all attenuating immune responses to TAAs. For instance, NSCLC cells frequently upregulate PD‐L1 to evade cytotoxic CD8⁺ T cell responses, with PD‐L1 expression correlating with poor clinical outcomes.^[^ ^166^ ^]^ In contrast, neoantigens—derived from tumor‐specific mutations such as KRAS G12C and EGFR T790M—are uniquely immunogenic. Delivering neoantigen libraries via mRNA enables the development of personalized cancer vaccines. For example, one study demonstrated that autologous dendritic cells pulsed with neoantigen mRNA elicited robust CD8⁺ T cell responses in melanoma patients.^[^ ^167^ ^]^ Co‐delivery of anti‐PD‐L1 mRNA alongside tumor antigen mRNA has further enhanced T cell activation. In a murine lung adenocarcinoma model, lipid nanoparticles (LNPs) co‐encapsulating OVA antigen and anti–PD‐L1 mRNA achieved a 65% reduction in tumor volume compared to antigen monotherapy, with increased CD8⁺ T cell infiltration and decreased Treg frequency.^[^ ^168^ ^]^

Layered polymer–hyaluronic acid hybrid nanoparticles (LPHNPs), composed of a PLGA core and a hyaluronic acid‐modified lipid shell, were designed to target CD44‐overexpressing NSCLC cells. When co‐loaded with KRAS G12C neoantigen mRNA and CTLA – 4 siRNA (for Treg depletion), these particles exhibited enhanced tumor accumulation and robust activation of antigen‐specific T cells, resulting in 70% inhibition of tumor growth in KRAS‐mutant lung cancer models.^[^ ^169^ ^]^

Despite these promising preclinical outcomes, several challenges remain. Accurate identification of neoantigens requires whole‐exome sequencing and bioinformatic analysis for each patient, which complicates clinical translation. Additionally, careful dose optimization of immune checkpoint modulators is critical to prevent autoimmune toxicity. Future research should focus on integrating tumor‐specific antigens with immunomodulatory components to advance mRNA therapies from nonspecific cytotoxic approaches to highly personalized, immune‐driven precision medicine for lung cancer.

### 5.5 Treatment of COPD

COPD is a major contributor to global morbidity, mortality, and healthcare burden. It is primarily caused by exposure to harmful inhaled particles, such as tobacco smoke and environmental pollutants.^[^ ^170^ ^]^ Current treatment strategies include bronchodilators, anti‐inflammatory drugs, and anti‐infective agents. Although these treatments alleviate symptoms and prevent exacerbations, they have limited effectiveness in reversing airway remodeling or improving airflow obstruction over time.^[^ ^171^ ^]^ The primary goals of pharmacological treatment are to minimize symptoms, enhance exercise tolerance, and reduce the risk of exacerbations. However, no pharmacological therapy has demonstrated a significant impact on improving survival in patients with COPD.^[^ ^172^ ^]^

Recent evidence^[^ ^173^ ^]^ highlights the role of macrophage‐derived EVs in COPD. These EVs carry inflammatory molecules, such as cytokines, chemokines, adhesion molecules, and proteases, which are implicated in COPD's pathological processes. As both producers and targets of EVs, macrophages are essential for facilitating interactions between epithelial cells and macrophages. In acute exacerbations of COPD (AECOPD), exosomes have been associated with the inflammatory process, correlating with plasma levels of soluble tumor necrosis factor receptor‐1, interleukin‐6, and C‐reactive protein. Detecting these particles in patients with AECOPD could provide insights into disease progression and inform targeted therapies to improve clinical outcomes.

**Figure 13:**
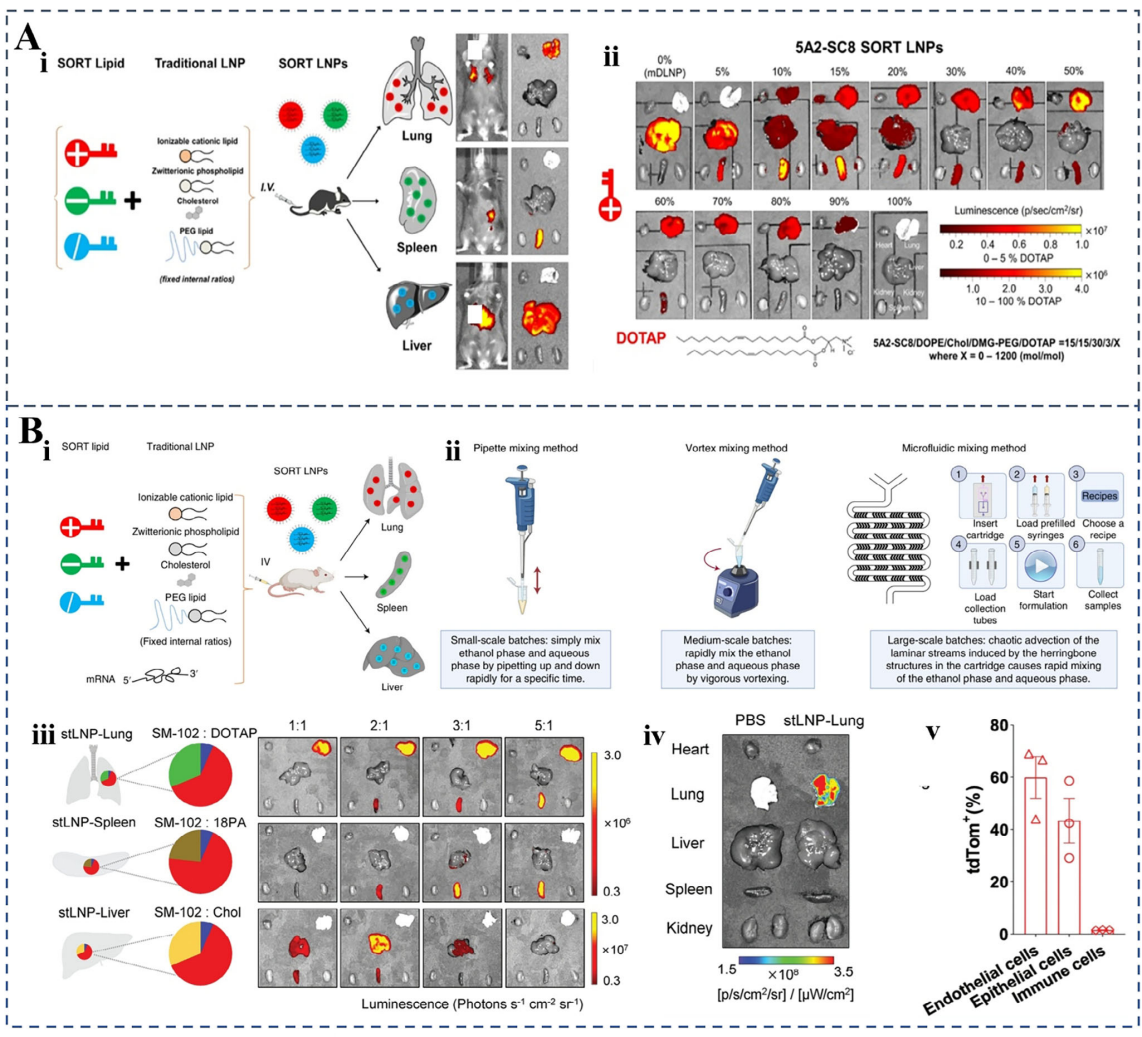
Selective Organ Targeting (SORT) technology allows for the systematic and predictable engineering of lipid nanoparticles (LNPs) to achieve precise delivery of mRNA to specific organs. A) Incorporating an additional component, designated as a SORT molecule, into conventional lipid nanoparticles (LNPs) systematically modifies their in vivo delivery characteristics and enables tissue‐specific targeting, which is dependent on the proportion and physicochemical attributes of the SORT molecule(i). Permanent cationic lipids (DOTAP) systematically transfer luciferase protein expression from the liver to the spleen to the lung as a function of DOTAP percentage (ii). Reproduced with permission.^[^ ^7^ ^]^ Copyright 2020 Springer. B) The addition of SORT molecules to conventional four‐component LNP consisting of ionizable cationic lipids, amphiphilic phospholipids, cholesterol, and PEG lipids systematically altered the in vivo delivery profiles of the resulting five‐component SORT LNP, enabling tissue‐specific delivery of mRNA to the liver, lung, and spleen of mice after intravenous injection(i). Three mixing methods were used to prepare the presented SORT LNP formulations: pipette mixing, vortex mixing, and microfluidic mixing(ii). Modulation of target lipids alters stLNP organ targeting specificity (iii). Reproduced with permission.^[^ ^181^ ^]^ Copyright 2023 Springer. Ex vivo imaging and quantification of tdTom fluorescence in key organs(iv). Flow cytometry analysis showed that ≈60% of endothelial cells, 40% of epithelial cells, and 2% of immune cells were edited(v). Reproduced with permission.^[^ ^185^ ^]^ Copyright 2024 Wiley.

Recent advancements in drug delivery systems hold promise for improving COPD management. LNPs conjugated to polyPBAEs and lipid molecules using microfluidic devices enable efficient delivery of mRNA and DNA through the lungs or systemically.^[^ ^72^ ^]^ Deng et al. developed amphiphilic PBAE nanoparticles targeting lung endothelial cells, offering efficient delivery. By adjusting the ratio of two alkyl chains in the nanoparticle backbone and incorporating PEG at the ends, transfection efficiency can be significantly optimized.^[^ ^174^ ^]^ The fluorination of these nanoparticles further enhances their efficiency. These customized PBAE nanoparticles open new therapeutic possibilities for COPD. Encapsulation of drugs within LNPs enhances their local concentration and retention time in the lungs (**Figure** **13**A,(i–v)), increasing therapeutic efficacy.^[^ ^175^ ^]^ Surface modifications, such as polyethylene glycolization (PEGylation), further improve the ability of LNPs to permeate airway mucus. De et al. demonstrated that PEGylated liposomes are more permeable in pathological sputum obtained from patients with COPD.^[^ ^176^ ^]^ Altering the surface properties of LNPs enhances their ability to evade airway clearance mechanisms, prolong their lung retention, and sustain the release of their therapeutic payload over an extended period, resulting in higher local drug concentrations and improved treatment outcomes.^[^ ^177^ ^]^ Inhaled LNP‐based therapies hold significant potential to transform COPD treatment by enhancing drug delivery efficiency, reducing side effects, and improving disease management. These advancements could also alleviate the global healthcare burden of COPD and other chronic lung diseases. Continued research and development in nanoparticle technology and drug delivery systems are essential for successfully translating these innovative therapies into clinical practice.

### 5.6 Treatment Acute Respiratory Distress Syndrome (ARDS)

ARDS is a heterogeneous condition characterized by acute inflammatory injury to the lungs, altered vascular permeability, loss of ventilated tissue, bilateral infiltrates, and refractory hypoxemia, complicating the development of effective treatments.^[^ ^178^ ^]^ Current medications include corticosteroids, statins, interferons, NSAIDs, anti‐CD14 drugs, and beta‐agonists. Although these treatments provide transient relief, they often have significant side effects. For instance, corticosteroids can lead to hyperglycemia, immunosuppression, and muscle weakness.

mRNA therapeutics offer promising possibilities for ARDS treatment owing to their potential for precise targeting and controlled protein expression. Achieving effective mRNA delivery requires the use of Selective Organ Targeting (SORT) delivery platforms, which enable specific delivery to lung tissue^[^ ^7^ ^]^ (Figure 13A,(i,ii)). SORT technology enables precise targeting of the affected region, thereby improving disease management and reducing side effects associated with systemic protein expression.

Cheng et al. used SORT technology to modulate the proportion of cationic lipids, such as DOTAP, within LNPs to achieve lung‐specific delivery.^[^ ^7^ ^]^ The core design of SORT‐LNPs involves substituting the predominantly ionizable lipids found in conventional LNPs with a high proportion (>50%) of cationic lipids, complemented by adding a specific SORT molecule. Depending on the proportion of SORT molecules incorporated and the resulting biophysical properties, this component modifies the in vivo RNA delivery profile and enables tissue‐specific gene delivery and editing. Targeting lung tissue is facilitated by the electrostatic interaction between positively charged LNPs and negatively charged surface molecules, such as acetylheparan sulfate proteoglycans on pulmonary vascular endothelial and alveolar epithelial cells. Due to their positive surface charge, SORT‐LNPs inhibit the adsorption of apolipoprotein E (ApoE) and instead enrich lung‐specific proteins, such as surfactant protein A, SP‐A, within the lung tissue microenvironment. This enhancing targeting. Cationic LNPs are internalized by lung endothelial and alveolar epithelial cells through lattice protein‐mediated endocytosis.^[^ ^179^ ^]^ In experiments involving intravenous injections in mice, SORT‐LNPs demonstrated a 10‐ to 20‐fold increase in mRNA delivery efficiency to the lungs compared to conventional LNPs, including those used in the Moderna and Pfizer vaccines. The study provided strong evidence of tissue‐specific delivery and demonstrated the adaptability of SORT to various nanoparticle systems. It also introduced a framework for the rational design of LNPs for targeting therapeutically relevant cells. However, high proportions of cationic lipids may elicit local inflammatory responses. To address this issue, chemical modifications are needed to balance delivery efficacy with biocompatibility, such as incorporating degradable linkers.^[^ ^180^ ^]^

The efficient targeting and potential toxicity of SORT‐LNPs have driven technological advancements. Based on these advancements, Wang et al. expanded on this approach by developing a refined SORT method that allows controlled delivery of nucleic acids to specific organs—including the liver, lung, and spleen—through intravenous administration.^[^ ^181^ ^]^ Multiple SORT‐LNP classes were generated using pipetting, vortexing, and microfluidic mixing techniques, followed by thorough physical characterization and evaluation of mRNA delivery both in vitro and in vivo. This method effectively enhanced organ‐specific nucleic acid delivery (Figure 13B,(i–ii)).

Wang et al.’s multi‐organ delivery strategy provided a new idea for precision targeting, while Yuan et al. further advanced this technology by developing a three‐component LNP platform optimized for targeted mRNA delivery to the lung and liver.^[^ ^181^ ^]^ Their strategy included engineering the mRNA scaffold with unique microRNA target sites to enable cell‐specific control of protein translation within the target tissues. The optimized formulation—stLNP‐Lung (SM‐102/DOTAP/PEG‐DMG) at a ratio of 65.7/32.8/1.5 was specifically directed to the lung. Flow cytometry analysis revealed that this formulation successfully edited ≈60% of endothelial cells, 40% of epithelial cells, and 2% of immune cells, demonstrating broad and efficient mRNA delivery across key lung cell populations (Figure 13B,(iii–v)).

Sun et al. demonstrated that DOTAP‐added LNPs delivering soluble programmed death ligand PD‐L1 (sPD‐L1) mRNA enabled specific expression in lung tissues, providing in situ immunosuppression in ARDS‐affected lungs and offering a novel therapeutic approach for acute inflammation.^[^ ^182^ ^]^ McCarthy et al. showed that IκBα‐SR mRNA reduced arterial carbon dioxide (pCO2) levels and lowered the lung wet‐to‐dry ratio.^[^ ^183^ ^]^ This was achieved by complexing mRNA encoding IκBα‐SR or SOD3 with cationic lipids and delivering it through a vibrating mesh nebulizer to cell cultures or rats with *E. coli* pneumonia. Additionally, SOD3 mRNA enhanced static lung compliance, improved the alveolar‐arterial oxygen gradient (AaDO2), and decreased the bacterial load in bronchoalveolar lavage (BAL). These findings highlight the potential of inhaled mRNA therapy as a novel and effective approach for treating ARDS. Some lung diseases can be modeled first to predict and better target the disease.^[^ ^184^ ^]^

## 6 Safety and Immune Response

Upon entering a cell, mRNA can be recognized by pattern recognition receptors, such as RIG‐I‐like receptors in the cytoplasm, which detect specific structural features of mRNAs.^[^ ^188^ ^]^ Overactivation of these PRRs, triggered by structural, modification, or purity issues in mRNA, may induce a robust intrinsic immune response. This can result in the excessive release of inflammatory factors such as IFN, which disrupt normal cellular functions, including protein synthesis, proliferation, and differentiation.^[^ ^31^ ^]^ Additionally, unmodified mRNAs are highly immunogenic due to their nucleotide structure and potential impurities, which are recognized as foreign pathogen‐associated molecular patterns. This recognition activates immune cells, such as dendritic cells and macrophages, leading to systemic inflammation that may manifest as fever and malaise.^[^ ^189^ ^]^ LNPs, widely used as mRNA delivery vehicles, pose potential safety concerns. Cationic lipids in LNPs may exhibit cytotoxicity by interfering with cell membrane fluidity and protein activity. Furthermore, LNP‐mRNA complexes may accumulate in non‐target tissues, such as the liver and spleen, triggering inflammatory responses and impairing normal organ functions. Similarly, polymer‐based delivery systems can present biocompatibility challenges. Specific polymers are difficult to degrade in vivo, potentially causing chronic inflammation, whereas impurities introduced during synthesis may provoke immune reactions or cytotoxicity.

Modifications to mRNA and delivery systems are critical to improving the safety of mRNA therapy. Chemical modifications, such as replacing uracil (U) with pseudouridine (Ψ), reduce PRR recognition, thereby decreasing inflammatory factor release and immune cell activation.^[^ ^190^ ^]^ These modifications also enhance mRNA stability, enabling more effective therapeutic action with fewer immune‐mediated side effects. Optimization of LNP properties further mitigates risks. Adjusting lipid types, proportions, and structures can modulate nanoparticle interactions with cell membranes.^[^ ^191^ ^]^ For instance, using phospholipids with varying hydrophilicity can enhance membrane interactions. Ionizable lipids, which change charge properties in specific pH environments, improve cellular uptake and facilitate endosomal escape.^[^ ^192^ ^]^ PEG modifications extend nanoparticle circulation time by reducing renal and MPS clearance.^[^ ^193^ ^]^ Targeting ligands can be tailored to specific cell markers, such as sodium‐dependent phosphate transport protein 2b (NaPi2b) on alveolar epithelial cells.^[^ ^194^ ^]^ Delivery systems achieve higher specificity by incorporating antibodies, peptides, or small molecules as targeting ligands, enhancing pharmacokinetics and biodistribution. This approach ensures effective mRNA concentrations in target tissues, minimizing off‐target effects and maximizing therapeutic efficacy.

Biomaterials play a crucial role in mRNA therapeutics by protecting mRNA and enhancing delivery efficiency. However, these materials’ intrinsic properties may also trigger adverse effects, such as immune activation or toxicity. For instance, gold nanomaterials (AuNPs) are chemically inert and prone to long‐term accumulation in organs such as the liver, spleen, and lungs.^[^ ^195^ ^]^ AuNPs smaller than 10 nm can cross the blood–lung barrier and accumulate in the endoplasmic reticulum of alveolar type II cells, interfering with surfactant synthesis. Prolonged exposure has been shown to double malondialdehyde (MDA) levels in lung tissue, indicating increased lipid peroxidation. After PEG‐SH coatings are cleared, exposed Au^3^⁺ ions bind to glutathione, which depletes intracellular antioxidants and reduces alveolar macrophage phagocytosis by 40%. Although AuNPs smaller than 5 nm have a clearance half‐life of ≈24 h, lung retention rates remain at 10–15%. Larger particles (>20 nm) are primarily cleared via the hepatobiliary pathway, requiring 3–6 months for complete elimination. Designing a degradable gold–thiol dynamic covalent network that dissociates AuNPs into nontoxic gold clusters (<2 nm) after mRNA delivery to mitigate these issues. Additionally, magnetic targeting technologies, such as external magnetic fields, can be used to direct particles to the liver and spleen for central clearance^[^ ^196^ ^]^ .

MSNs, which are composed of a silica–oxygen backbone, degrade very slowly under biological conditions. At a lysosomal pH of 4.5, they degrade at a rate of only 0.1% per day. Long‐term accumulation has been associated with chronic inflammation and fibrosis.^[^ ^197^ ^]^ Animal studies show that 12 months after intravenous administration, 20–30% of MSNs remain sequestered in lung macrophages, accompanied by local granuloma formation and increased collagen deposition. Although some MSNs can be eliminated via renal filtration, their pulmonary clearance half‐life exceeds 180 days. MSNs are primarily transported through the lymphatic system to mediastinal lymph nodes, resulting in persistent accumulation.^[^ ^198^ ^]^ Introducing breakable silane bonds (e.g., ester or amino group modifications) can increase degradation rates by 5–10‐fold, accelerating clearance. Additionally, surface grafting with polyethylene glycol (PEG5000) has been shown to reduce protein adsorption and macrophage uptake, lowering uptake rates from 40% to 15%.^[^ ^199^ ^]^

The binding of cationic lipids to erythrocyte membranes can easily cause hemolysis. When the lipid/mRNA charge ratio (N/P) exceeds 8, hemolysis surpasses the 5% safety threshold by a significant amount. In the pulmonary vasculature, high‐charge‐density lipids can damage the tight junctions between endothelial cells. This disruption can result in protein leakage and pulmonary edema^[^ ^200^ ^]^ Furthermore, cationic lipids can adsorb endotoxins, such as LPS. These endotoxins can indirectly activate immune cells. This activation can cause the serum IL‐6 levels to increase over tenfold, potentially triggering systemic inflammatory response syndrome (SIRS).^[^ ^201^ ^]^ Cationic lipids are primarily broken down into fatty acids by liver microsomal enzymes. However, the residual lipids in the lungs are slowly metabolized by alveolar macrophages, with a half‐life exceeding 30 days. This persistent presence leads to the ongoing release of inflammatory signals. One solution is to substitute cationic lipids with electroneutral ionizable ones (e.g., SM‐102). These ionizable lipids only become protonated in acidic endosomes (at pH < 6.5). Substituting cationic lipids with electroneutral ionizable lipids reduces serum IL‐6 levels by 70% while preserving transfection efficiency. Additionally, keeping the N/P ratio at or below 5 mitigates the toxicity of free lipids.^[^ ^202^ ^]^

Overactivation of the proton sponge effect by polyamidoamine (PAMAM) dendrimers can result in lysosomal rupture and the release of cathepsin B, triggering apoptotic pathways. Although PAMAM is partially cleared via glomerular filtration, it tends to accumulate in the liver and spleen, with only ≈5% excreted in urine. To overcome this limitation, incorporating disulfide‐ or hydrazone‐cleavable bonds into the polymer backbone allows PAMAM to depolymerize into less toxic oligomers within the intracellular reducing environment.^[^ ^203^ ^]^  Furthermore, surface modification with sialic acid, which mimics the glycocalyx, can reduce complement activation by up to 80%, thereby improving biocompatibility.

Addressing the safety and immunogenicity concerns of mRNA therapy requires a dual approach that combines molecular modifications with advanced delivery system engineering. These innovations are crucial for minimizing adverse effects while enhancing the precision and therapeutic efficacy of mRNA‐based treatments. Ongoing research and continuous refinement of these strategies are essential to fully unlock the clinical potential of mRNA therapeutics.

## 7 Challenges and Perspectives of mRNA Delivery by Biomaterials

mRNA has emerged as a promising therapeutic tool for a wide range of lung diseases, offering advantages such as directing the expression of therapeutic proteins, low immunogenicity, long‐term stability, and efficient translational capacity. Various biomaterials have been engineered to facilitate mRNA delivery, each possessing unique characteristics that protect mRNA, promote cellular uptake, enable targeted delivery, and control release. These features collectively improve the delivery efficiency and therapeutic efficacy of mRNA. However, significant challenges remain. The respiratory mucosal surface of the lungs, covered with cilia and mucus, forms a physical barrier that hinders mRNA delivery to the alveolar region. Mucus may adsorb mRNA carriers, delaying their delivery to target cells and influencing both the rate and extent of drug absorption. Although biomaterials can be engineered with specific surface properties to enhance cellular uptake, the efficiency of this process remains limited. Different lung cell types exhibit varying capacities for mRNA carrier uptake, potentially leading to uneven drug distribution and reduced therapeutic efficacy. Once inside the cell, mRNA is often encapsulated within endosomes. The stability of the endosomal membrane can prevent efficient escape of the mRNA into the cytoplasm, where translation occurs. Without successful endosomal escape, the therapeutic potential of the mRNA cannot be achieved. Additionally, unmodified mRNA is highly immunogenic, potentially triggering systemic inflammatory responses, such as fever and malaise. Specific biomaterials, including lipid and polymer nanoparticles, can also provoke immune and inflammatory responses owing to their composition or properties. Strategies to overcome these challenges have primarily concentrated on enhancing the efficiency of mRNA delivery and refining its targeting specificity. Advanced nebulization equipment and techniques, such as optimized nebulizers and adjusted nebulization buffers, can enhance the stability of mRNA during nebulization, reducing aggregation and loss. Directed delivery to particular cell populations can minimize off‐target effects and enhance therapeutic outcomes. The development of novel polymer materials or specific polymer modifications has also shown promise in improving the performance of polymer nanoparticles. Hybrid nanoparticles, integrating the benefits of lipids and polymers, can optimize their composition and structure to achieve improved drug encapsulation, controlled release, and targeted delivery. Additionally, the properties of delivery vehicles, such as lipid, polymer, and hybrid nanoparticles, can be further refined to increase stability, extend in vivo circulation time, and enhance fusion with cell membranes, ensuring efficient cellular delivery and sustained mRNA expression. Despite progress in mRNA delivery using biomaterials for diseases such as PF, asthma, pneumonia, lung cancer, COPD, and ARDS, several challenges remain. These include obstacles in the delivery process, risks of immune stimulation and inflammatory responses, and the need for strategies to improve delivery efficiency and targeting. Additionally, strategies to achieve long‐term stable expression are required, along with comprehensive toxicity assessment and long‐term safety evaluation. Future research should prioritize refining delivery systems, improving targeting strategies, and addressing long‐term safety concerns. The successful exploitation of the therapeutic potential of mRNA technologies for lung diseases hinges crucially on these efforts.

## 8 Conclusion 

Lung disease remains a significant global health challenge, with current treatments offering limited effectiveness. mRNA therapy represents a transformative approach, bringing new possibilities for lung disease treatment. However, challenges such as mRNA stability, delivery efficiency, and cell specificity continue to hinder its clinical application. Biomaterials, as carriers, play a crucial role in addressing these challenges by protecting mRNA, enhancing delivery efficiency, and enabling targeted delivery. Despite their potential, biomaterials for mRNA delivery remain in the developmental stage and require further extensive research and optimization. Future research should prioritize addressing existing challenges, including improving the performance and safety of biomaterials, enhancing the delivery efficiency and targeting specificity of mRNA, and minimizing immune response risks. In addition, understanding the structure–function relationship of biomaterials and their interactions with cells and tissues in physiological environments will be critical. This knowledge will expedite the development of innovative, efficient, and safe mRNA delivery systems, thereby accelerating their clinical translation and expanding their applications. The continued advancement of mRNA delivery systems holds the promise of expanding therapeutic options for patients with lung disease, offering hope and paving the way for innovative, effective treatments in the future.

## Conflict of Interest

The authors declare no conflict of interest.

## Author Contributions

Q.G. performed validation and wrote the original draft. H.X. developed the methodology and wrote, reviewed, and edited the final draft. Z.L. performed the investigation and data curation. J.R. worked with the software. L.Z. visualized the study and performed data curation. C.Z. performed validation. L.L. administered the project and acquired funds. L.S. administered the project and acquired funds.

## Ethics Approval and Consent to Participate

No human or animal subjects are investigated in this review, and informed consent is not applicable.
